# BCL::Fold - *De Novo* Prediction of Complex and Large Protein Topologies by Assembly of Secondary Structure Elements

**DOI:** 10.1371/journal.pone.0049240

**Published:** 2012-11-16

**Authors:** Mert Karakaş, Nils Woetzel, Rene Staritzbichler, Nathan Alexander, Brian E. Weiner, Jens Meiler

**Affiliations:** Department of Chemistry, Center for Structural Biology, Vanderbilt University, Nashville, Tennessee, United States of America; University of Oxford, United Kingdom

## Abstract

Computational *de novo* protein structure prediction is limited to small proteins of simple topology. The present work explores an approach to extend beyond the current limitations through assembling protein topologies from idealized α-helices and β-strands. The algorithm performs a Monte Carlo Metropolis simulated annealing folding simulation. It optimizes a knowledge-based potential that analyzes radius of gyration, β-strand pairing, secondary structure element (SSE) packing, amino acid pair distance, amino acid environment, contact order, secondary structure prediction agreement and loop closure. Discontinuation of the protein chain favors sampling of non-local contacts and thereby creation of complex protein topologies. The folding simulation is accelerated through exclusion of flexible loop regions further reducing the size of the conformational search space. The algorithm is benchmarked on 66 proteins with lengths between 83 and 293 amino acids. For 61 out of these proteins, the best SSE-only models obtained have an RMSD100 below 8.0 Å and recover more than 20% of the native contacts. The algorithm assembles protein topologies with up to 215 residues and a relative contact order of 0.46. The method is tailored to be used in conjunction with low-resolution or sparse experimental data sets which often provide restraints for regions of defined secondary structure.

## Introduction

Understanding of protein function and mechanics is facilitated by and often depends on the availability of structural information. The Protein Data Bank (PDB), as of April 2011, holds 66,726 protein structure entries, 87% determined by X-Ray crystallography and 12% determined by Nuclear Magnetic Resonance (NMR) spectroscopy, and the remaining 1% determined by Electron microscopy and hybrid methods [1], [2]. The millions of protein sequences revealed by genome projects necessitate utilization of computational methods for construction of protein structural models. Comparative modeling utilizes structural information from one or more template proteins with high sequence similarity to the protein of interest to construct a model. As the PDB grows and the number of proteins with an existing suitable template of known structure increases, this method is unarguably most important [3].

However, despite impressive advancements in the combination of experimental protein structure determination techniques [4], [5] with comparative modeling [6], entire classes of proteins remain underrepresented in the PDB as they evade crystallization or are unsuitable for NMR studies; e.g. membrane proteins [7] and proteins that only fold as part of a large macromolecular assembly [8], [9]. Such proteins more frequently adopt topologies not yet represented in the PDB such that the current structural knowledge fails to encapsulate necessary information to represent all protein families and folds expected to be found in nature [10]. In such situations *de novo* methods for prediction of protein structure from the primary sequence alone can be applied.

### De Novo Protein Fold Determination is Possible for Smaller Proteins of Simple Topology

*De novo* protein structure prediction typically starts with predicting secondary structure [11], [12], [13], [14] and other properties of a given sequence such as β-hairpins [15], disorder [16], [17], non-local contacts [18], domain boundaries [19], [20], [21], and domain interactions [22], [23]. System-learning approaches such as artificial neural networks (ANN), hidden Markov models (HMM), and support vector machines (SVM) are most commonly used in this field [24], [25].

This preparatory step is followed by the actual folding simulation. Rosetta, one of the best performing *de novo* methods, follows a fragment assembly approach [26], [27], [28]. For all overlapping nine- and three- amino acid peptides of the sequence of interest, conformations are selected from the PDB by agreement in sequence and predicted secondary structure. Rosetta is capable of correctly folding about 50% of all sequences with less than 150 amino acids [29].

Chunk-Tasser is another fragment assembly method for de novo structure prediction that was one of the best performing methods in the CASP8 experiment [30]. This method generates chunks, three consecutive secondary structure elements (SSEs) connected by two loops, using nine- and three- residue fragments. The final models are built by using these chunks as the starting point coupled with a minimization process that also utilizes threading and distance restraint predictions [31].

### For Small Proteins with Less than 80 Amino Acids Models can Sometimes be Refined to Atomic-detail Accuracy

**Figure 1:**
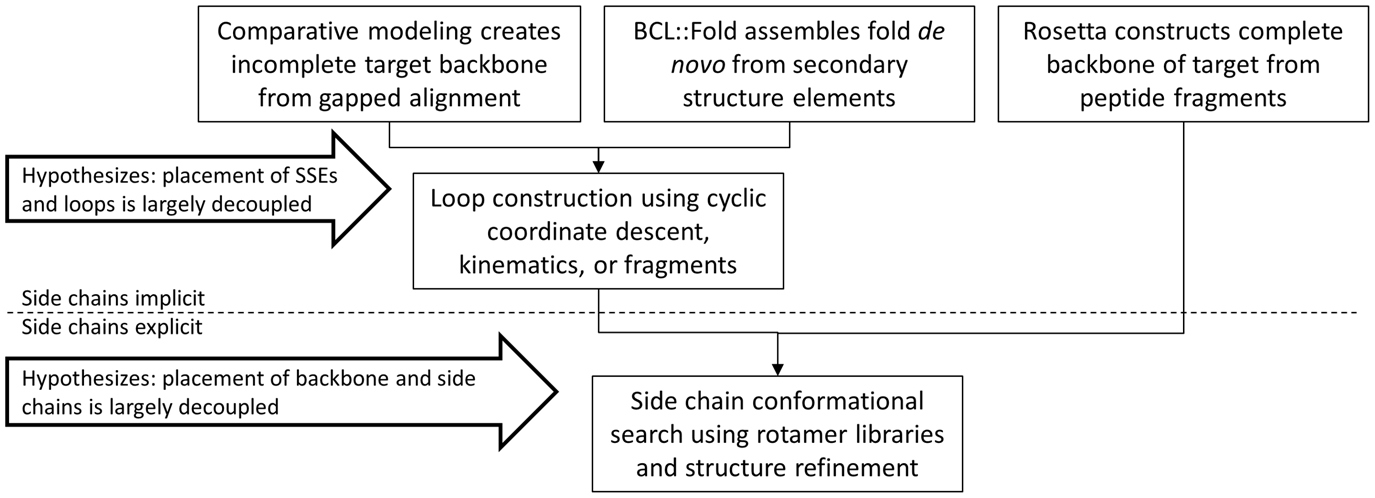
Comparison of comparative modeling, BCL::Fold and Rosetta. In comparative modeling the protein backbone is constructed partially from a target-template alignment, followed by loop construction and side chain building. On the other hand, *de novo* methods, such as Rosetta, only take advantage of the decoupling of backbone placement and the side chain building. BCL::Fold also decouples the construction of loops from assembly of secondary structure elements, similar to comparative modeling. Although disconnecting these steps makes computation more feasible by splitting the total search space into manageable portions, they are not absolute and in order to address these issues SSE placement has to be refined before loop building and side chain construction. While side chain conformations are not explicitly added to the model in the early stages of *de novo* structure prediction, they are implicitly represented throughout the process through knowledge-based potentials.

During the folding simulation, most *de novo* methods use a reduced protein representation that excludes side chain degrees of freedom to simplify the conformational search space and potential. The fastest and most accurate algorithms to add side chains in order to build atomic detail models rely on sampling likely conformations of amino acid side chains, so-called rotamers [32], [33], [34]. At this stage, the backbone of flexible loop regions can be further refined, in Rosetta by a combination of fragment insertions, side chain repacking, and gradient minimization. In the CASP6 experiment, Rosetta was able predict *de novo* the structure of a small α-helical protein to a resolution of 1.59Å [26]. Following this success, Bradley and co-workers showed comprehensively that high resolution backbone structure prediction facilitates the correct placement of side chains and thus *de novo* high resolution structure elucidation for small proteins [35]. Note that the refinement of backbone conformations and construction of side chain coordinates aligns with most comparative modeling protocols [36], [37] (Figure 1). These algorithms model gaps and insertions using loop closure algorithms that use analytical geometry [38], molecular mechanics [39], or loop libraries from the PDB [40] before entering the refinement process. Thereby both approaches – *de novo* structure prediction and comparative modeling – share the decoupling of the construction of backbone and side chain coordinates. This procedure relies on the hypothesis that accurately placed backbone coordinates define the side chain conformations [33] (Figure 1).

### Progress is Stalled by Inefficient Sampling of Large and Complex Topologies

*De novo* methods perform well only for small proteins, because the conformational search space increases rapidly as the protein gets larger. Despite simplified representation of proteins that omit side chain degrees of freedom, sampling the correct topology remains the major bottleneck for folding large proteins. Sampling is complicated for large proteins not only by size, but also by a larger number of non-local contacts, i.e. interactions between amino acids that are far apart in sequence. More of these interactions contribute to protein stability and are therefore important to sample in order to find the correct topology. At the same time, when folding a continuous protein chain each of these contacts complicates the search as conformational changes between the two amino acids in contact require coordinated adjustment of multiple phi, psi angles to not disrupt the contact. To quantify the number of such non-local contacts the relative contact order (RCO) of a protein was defined which is the average sequence separation of residues “in contact”, i.e. having their C_β_ atoms (H_2α_ for Glycine) within 8Å [41], [42] normalized by sequence length. As the RCO increases above 0.25 (i.e. the average separation of two residues that are in contact is 25% of the sequence length), the success rate of *de novo* prediction drops drastically [43]. Also, the geometry of non-local interactions and β-strand pairings in particular is often inaccurate as relative placement of the SSEs cannot be optimized independently from the connecting amino acid chain. This limitation must be overcome for *de novo* methods to be successfully applied to larger proteins. Interestingly, contact order correlates also with protein folding rates [44] suggesting that the sampling of non-local contacts is the rate-limiting step in protein folding.

### De novo Protein Structure Prediction Optimally Leverages Limited Experimental Datasets for Proteins of Unknown Topology

Experimental structural data that becomes available for proteins of unknown topology are often limited, i.e. sparse or low in resolution. Typically, these limited data sets focus on and are more readily available for backbone atoms in ordered secondary structure elements. For example, cryo-Electron Microscopy and X-Ray crystallography yield medium resolution density maps of 5–10 Å where secondary structure can be identified but loop regions and amino acid side chains remain invisible [45], [46], [47], [48], [49]. NMR and EPR spectroscopy yield sparse datasets due to technological or resource limitations [49], [50], [51], [52], [53], [54], [55]. Chemical cross linking coupled with mass spectrometry has also been shown to be applicable for protein structure determination at these low resolutions [56], [57], [58].

While *de novo* protein structure prediction is typically insufficient in accuracy and confidence to be applied to determine the structure of a protein without the help of experimental data, a series of manuscripts was published that demonstrated the power of such technologies to predict protein structures accurately at atomic-detail when combined with limited experimental data sets of different origin. Qian et al. previously demonstrated use of *de novo* structure prediction to overcome the crystallographic phase problem [59]. *De novo* methods have also been applied for rapid fold determination from unassigned NMR data [60] and structure determination for larger proteins from NMR restraints [61]. In addition, *de novo* structure prediction has also been coupled with EPR restraints [62], [63], [64] as well as cryo-EM [49]. Kahlkof et. al studied *de novo* structure prediction of laminin using distance restraints from natural cross-links revealed a structural similarity to galactose binding proteins [57], which was later confirmed when the structure was experimentally determined by X-Ray crystallography [65]. Numerous other studies have also harnessed the power of *de novo* structure prediction with experimental restraints [66], [67], [68].

The objective of the present work is to introduce an algorithm for protein folding with a novel approach of assembly of SSEs in three-dimensional space. This approach seeks to overcome size and complexity limits of previous approaches by discontinuing the amino acid chain in the folding simulation thereby facilitating the sampling of non-local contacts. Exclusion of loop regions focuses the sampling to the relative arrangement of rather rigid SSEs, limiting the overall search space. The approach can be readily combined with limited datasets which tend to restrain the location of backbone atoms in SSEs. It leverages established protocols for construction of loop regions and side chains to yield complete protein models (Figure 1). The decoupling of the placement of SSEs from the construction of loop regions relies on the hypothesis that accurate placement of SSEs will allow for construction of loop regions and subsequent placement of side chain coordinates, a hypothesis tested excessively in comparative modeling. This approach assumes further that the majority of the thermodynamic stabilization achieved through formation of the core of the protein is defined by interactions between SSEs and can therefore be approximated with an energy function that relies exclusively on scoring SSEs. This hypothesis has been tested in a companion manuscript “BCL::Score” (BCL - BioChemicalLibrary) in the same issue of this journal. Briefly, the scoring terms cover amino acid environment and pair-wise interaction; clash penalties prevent overlap; secondary structure is evaluated relative to the predicted probabilities; chain breaks are tested for being closable by a loop; the topology is evaluated by the radius of gyration, and SSE pairing and packing is represented by distance and dihedral angle. All potentials are derived using empirics from known protein structures.

Although the algorithm is in principle applicable to membrane protein structure prediction, changes in the sampling steps presented in the BCL::Fold algorithm as well as modifications of the energy terms in the composite energy functions of BCL::Score are required and are presently under investigation.

## Results and Discussion

In fragment assembly based approaches to *de novo* protein structure prediction, local contacts are sampled more efficiently than the non-local ones due to inherent restrictions imposed by the connectivity of the amino acid sequence. This restriction leads to one of the major challenge in *de novo* protein structure prediction – the sampling of complex topologies as defined by the abundance of non-local contacts and thus higher relative contact order (RCO) values [43]. Further, fragment based approaches spend a large fraction of time sampling the conformational space of flexible loop regions that contribute little to the stability of the fold. Therefore the accuracies of the methods deteriorate as the conformational search space gets larger, typically for proteins with more than 150 residues. In particular, β-strand interactions are often sampled insufficiently densely to arrive at the correct pairings with good geometries. As a result, regular secondary structure cannot be detected in the models giving them the well-known “spaghetti”-look. The score deteriorates hampering detection of the correct topology in a large ensemble of models.

### BCL::Fold is Designed to Overcome Size and Complexity Limitations in De Novo Protein Structure Prediction

**Figure 2:**
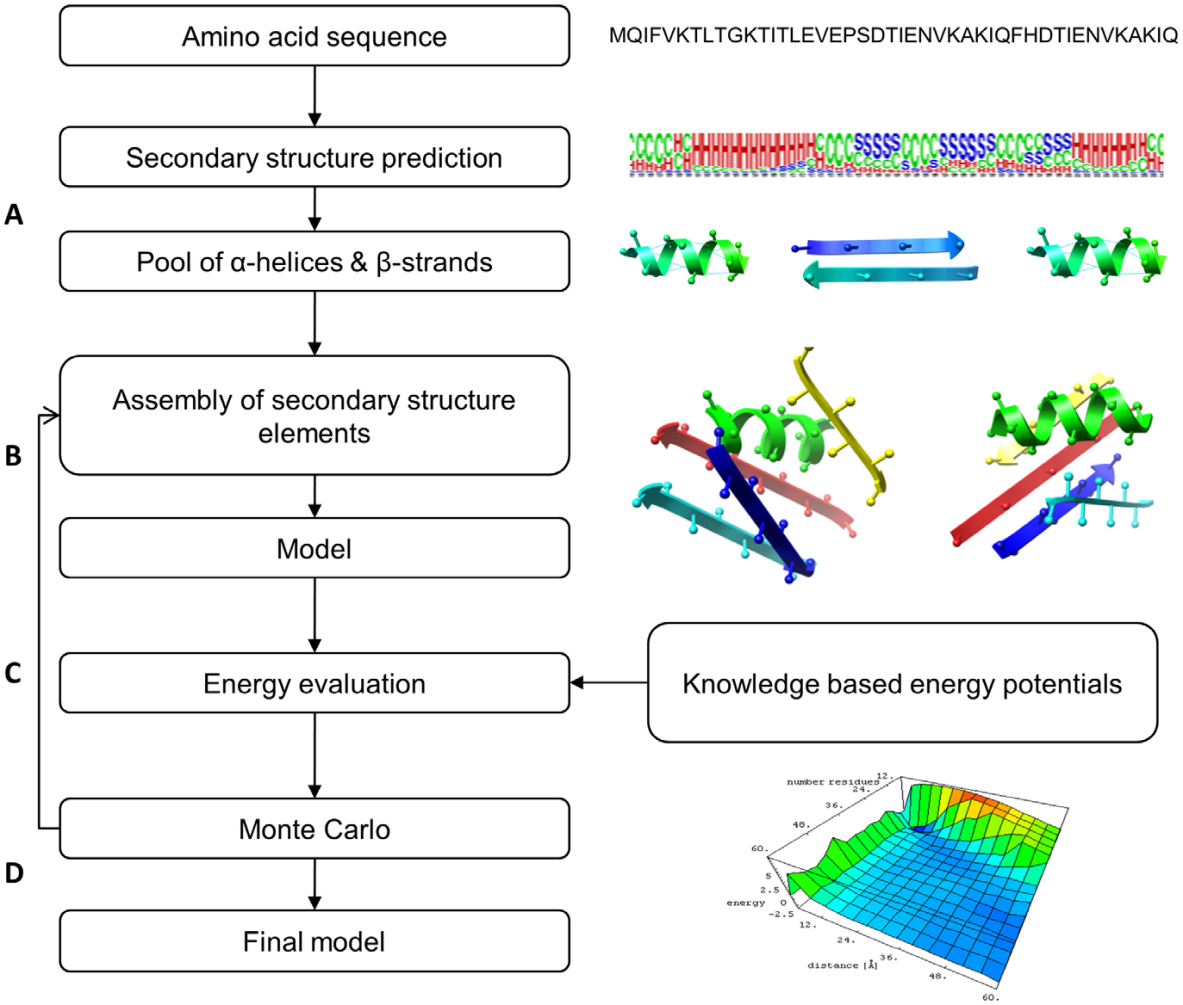
BCL::Fold protocol flowchart. (**A**) Generation of secondary structure element (SSE) pool. The secondary structure prediction methods, PSIPRED and JUFO, have been combined to achieve a consensus three state secondary structure prediction. For a given amino acid sequence, stretches of sequence with consecutive α-helix or β-strand predictions above a given probability threshold are identified as α-helical and β-strand SSEs and added to the pool of SSEs to be used in the assembly protocol. (**B**) Assembly of SSEs. The initial model only has a randomly picked SSE from the SSE pool. At each single iteration step, a move is picked randomly and applied to produce a new model. The details regarding utilized moves are given in the next panel. (**C**) Energy evaluation using knowledge based potentials**.** After each change, the model is evaluated using knowledge based potentials. These include loop, loop closure, amino acid environment, amino acid pair distance, amino acid clash, SSE packing, strand pairing, SSE clash, contact order and radius of gyration. (**D**) Monte Carlo Metropolis minimization. Based on the energy evaluation, models with lower energies than the previous model are accepted, while models with higher energy can be either accepted or rejected based on Metropolis criteria. The accepted models are further optimized, in case of rejected models, the minimization continues with the last accepted model. The minimization is terminated after either a specified total number of steps or a specified number of rejected steps in a row. The protocol consists of two such minimizations, one for assembly and one for refinement.

BCL::Fold assembles secondary structure elements (SSEs), namely α-helices and β-strands while not explicitly modeling loop conformations (Figure 2). Individual residues are represented by their backbone and C_β_ atoms only, (H_α2_ for Glycine). A pool of predicted SSEs is collected using a consensus of secondary structure prediction methods. A Monte Carlo Metropolis minimization with simulated annealing is used where models are altered by SSE-based moves (Table S1 and Table S2) and evaluated by knowledge-based energy potentials (Table S3). The reduced representation of proteins in BCL::Fold decreases the conformational search space that has to be sampled. Moving discontinued SSEs independently of each other accelerates sampling of non-local contacts. The knowledge-based scoring function employed by BCL::Fold is described in a companion manuscript in the same issue of this journal.

BCL::Fold was evaluated using a benchmark set of proteins collected using PISCES culling server. The set includes 66 proteins of lengths ranging from 83 to 293 residues with<30% sequence similarity. The set contains different topologies including 31 all α-helical, 16 all β-strand, and 19 mixed αβ folds (Table 1). The selected proteins have RCOs in the range of 0.12 to 0.47 with an average of 0.30±0.07. It should be noted that as proteins get larger, RCO values start decreasing (compare Figure S2). Therefore we introduced a normalized contact order measure NCO which is defined as the square of the contact order divided by sequence length and is largely independent of protein size.

**Table 1:** Benchmark set of proteins.

	FULL SEQUENCE						FILTERED SEQUENCE					
PDB id	N_aa_	N_sse_	N_α_	N_β_	CO	RCO	N_aa_	N_sse_	N_α_	N_β_	CO	RCO
1BGCA	174	7	7	0	67.75	0.39	108	5	5	0	81.94	0.47
1EYHA	144	8	8	0	33.59	0.23	107	8	8	0	36.48	0.25
1FQIA	147	9	9	0	44.35	0.30	90	9	9	0	46.87	0.32
1GAKA	141	7	7	0	57.17	0.41	96	6	6	0	51.38	0.36
1GYUA	140	10	2	8	34.86	0.25	63	8	0	8	32.51	0.23
1IAPA	211	11	11	0	60.11	0.28	123	9	9	0	77.40	0.37
1ICXA	155	13	6	7	47.25	0.30	103	10	3	7	46.52	0.30
1J27A	102	6	2	4	44.41	0.44	76	6	2	4	46.89	0.46
1JL1A	155	10	5	5	52.69	0.34	97	10	5	5	50.41	0.33
1LKIA	180	8	6	2	73.33	0.41	113	5	5	0	76.37	0.42
1LMIA	131	10	1	9	40.95	0.31	63	9	0	9	41.77	0.32
1OXJA	173	11	11	0	35.54	0.21	108	8	8	0	30.49	0.18
1OZ9A	150	10	5	5	34.00	0.23	101	9	5	4	37.53	0.25
1PBVA	195	10	10	0	30.84	0.16	128	10	10	0	30.06	0.15
1PKOA	139	13	3	10	44.12	0.32	58	9	0	9	43.50	0.31
1Q5ZA	177	11	11	0	40.42	0.23	77	6	6	0	46.33	0.26
1RJ1A	151	8	8	0	45.07	0.30	113	7	7	0	41.83	0.28
1T3YA	141	12	6	6	30.33	0.22	83	9	4	5	25.99	0.18
1TP6A	128	9	3	6	32.97	0.26	94	9	3	6	31.72	0.25
1TQGA	105	4	4	0	36.73	0.35	88	4	4	0	38.04	0.36
1TZVA	142	9	9	0	32.42	0.23	97	7	7	0	35.14	0.25
1UAIA	224	18	2	16	57.10	0.25	114	15	0	15	55.64	0.25
1ULRA	88	7	2	5	40.11	0.46	55	7	2	5	36.68	0.42
1VINA	268	16	16	0	51.29	0.19	156	12	12	0	51.04	0.19
1X91A	153	6	6	0	48.33	0.32	113	5	5	0	46.98	0.31
1XAKA	83	7	0	7	30.22	0.36	38	6	0	6	33.08	0.40
1XKRA	206	14	6	8	65.80	0.32	147	14	6	8	66.11	0.32
1XQOA	256	14	14	0	60.32	0.24	162	14	14	0	67.52	0.26
1Z3XA	238	14	14	0	36.63	0.15	129	13	13	0	32.88	0.14
2AP3A	199	7	7	0	53.65	0.27	156	5	5	0	55.95	0.28
2BK8A	97	10	1	9	35.03	0.36	47	7	0	7	30.67	0.32
2CWRA	103	9	0	9	35.71	0.35	60	8	0	8	33.53	0.33
2EJXA	139	10	3	7	41.78	0.30	107	10	3	7	38.38	0.28
2F1SA	186	12	12	0	30.75	0.17	115	12	12	0	35.40	0.19
2FC3A	124	10	6	4	47.78	0.39	80	9	5	4	51.27	0.41
2FM9A	215	10	10	0	58.23	0.27	153	9	9	0	59.69	0.28
2FRGP	106	11	2	9	36.63	0.35	64	9	0	9	33.94	0.32
2GKGA	127	11	6	5	32.56	0.26	80	10	5	5	32.51	0.26
2HUJA	140	4	4	0	50.34	0.36	99	4	4	0	53.84	0.38
2IU1A	208	11	11	0	42.10	0.20	126	10	10	0	43.75	0.21
2JLIA	123	8	4	4	30.25	0.25	69	8	4	4	29.23	0.24
2LISA	136	6	6	0	55.90	0.41	91	5	5	0	53.23	0.39
2OF3A	266	16	16	0	34.76	0.13	202	16	16	0	31.79	0.12
2OSAA	202	11	11	0	49.60	0.25	124	9	9	0	50.70	0.25
2QZQA	152	13	3	10	46.24	0.30	63	7	0	7	52.92	0.35
2R0SA	285	16	16	0	58.40	0.20	165	13	13	0	57.84	0.20
2RB8A	104	8	0	8	33.84	0.33	46	7	0	7	29.12	0.28
2RCIA	204	13	7	6	63.82	0.31	126	10	4	6	63.77	0.31
2V75A	104	5	5	0	32.84	0.32	65	5	5	0	34.26	0.33
2VQ4A	106	10	1	9	33.71	0.32	54	8	0	8	32.07	0.30
2WJ5A	101	7	1	6	31.44	0.31	42	6	0	6	28.26	0.28
2WWEA	127	8	5	3	34.86	0.27	69	7	4	3	35.10	0.28
2YV8A	164	14	1	13	59.67	0.36	79	12	0	12	56.88	0.35
2YXFA	100	9	1	8	32.85	0.33	46	7	0	7	31.37	0.31
2YYOA	171	14	1	13	50.72	0.30	66	12	0	12	58.41	0.34
2ZCOA	293	16	16	0	51.60	0.18	205	15	15	0	56.53	0.19
3B5OA	244	11	11	0	83.49	0.34	169	9	9	0	85.09	0.35
3CTGA	129	11	7	4	33.78	0.26	68	9	5	4	32.00	0.25
3CX2A	108	10	2	8	39.67	0.37	53	7	0	7	37.05	0.34
3FH2A	146	9	9	0	43.06	0.29	100	9	9	0	42.92	0.29
3FHFA	214	13	13	0	51.79	0.24	147	12	12	0	58.19	0.27
3FRRA	191	9	9	0	54.64	0.29	141	9	9	0	55.61	0.29
3HVWA	176	14	7	7	48.29	0.27	109	11	5	6	51.62	0.29
3IV4A	112	11	6	5	35.13	0.31	77	9	4	5	32.98	0.29
3NE3B	130	11	6	5	42.02	0.32	81	9	4	5	48.43	0.37
3OIZA	99	7	3	4	26.73	0.27	63	7	3	4	25.52	0.26

For each of the 64 proteins in the benchmark set, following are displayed: 4 letter code PDB id and 1 letter code chain id, number of amino acids (N_aa_), number of secondary structure elements(N_sse_), number of α-helices (N_α_), number of β-strands (N_β_), contact order (CO), relative contact order (RCO). The left section of the table identified as “original sequence” displays statistics for the full sequence protein, while the “filtered sequence” statistics are calculated only on amino acids that are found in secondary structure elements that satisfy the length criteria; at least 5 residues for α-helices and 3 residues for β-strands.

### Consensus Prediction of SSEs from Sequence to Create Comprehensive Pool for Assembly

The secondary structure prediction programs JUFO [13], [69] and PSIPRED [70] were used to create a comprehensive pool of predicted SSEs. Two methods are used to avoid deterioration of BCL::Fold performance if one of the methods fails. To further avoid dependence on potentially incorrect predicted secondary structure, we implement two strategies: a) the initial pool of SSEs contains multiple copies of one SSE having different length. In extreme cases of ambiguity this could be an α-helix predicted by one method and a β-strand predicted by the other or one long α-helix that overlaps with two short α-helices that span the same region. b) The length of SSEs is dynamically adjusted during the folding simulation in order to allow simultaneous optimization of protein secondary and tertiary structure [13]. Both strategies require a scoring metric that analyzes the agreement of a given set of SSEs with the predicted secondary structure. Before the actual folding simulation is started, a pool of likely SSEs is created using a rapid Monte Carlo Metropolis simulation. The scoring scheme and the pool generation are described in more detail in the methods section. SSEs predicted by this method are only added to the secondary structure pool if they satisfy the minimum length restrictions; five residues for α-helices and three residues for β-strands. Rationale for removal of very short SSEs is two-fold: a) the reduced accuracy of secondary structure prediction techniques for such short SSEs [71] and b) the limited contribution to fold stability expected from short SSEs (compare companion manuscript “BCL::Score – Knowledge based energy potentials for ranking protein models represented by idealized secondary structure elements”).

Table 2 depicts Q3 accuracies (a measure of the accuracy for predicting per residue secondary structure [72]), as well as the percentage of native secondary structures correctly predicted and the average shifts for the SSE pools of the 66 benchmark proteins using PSIPRED and JUFO secondary structure prediction. For this set of benchmark proteins, SSE pools generated using PSIPRED exhibited higher Q3 values (79.6% ±10.6 vs. 70.2% ±11.9) and higher native SSE recovery (96.1% ±6.4 vs. 90.3% ±10.7) when compared to JUFO. This trend is also observed for shift values (3.1±2.2 vs. ±4.3±2.8) which measure the sum of the deviations in first and last residues of the predicted SSEs when compared with native SSEs. Although PSIPRED has a better overall performance, a combined pool of PSIPRED and JUFO has the highest native SSE recovery (96.6%) and the lowest shift (2.7). Because the SSE pool is constructed in a pre-processing step, secondary structure prediction methods can be changed or SSEs can be manually adjusted if desired.

**Table 2:** Secondary structure pool statistics for the benchmark proteins.

pdb id	PSIPRED			JUFO			PSIPRED+JUFO	
	Q3	%found	shift	Q3	%found	shift	%found	shift
1BGCA	88.7	100.0	4.2	81.6	100.0	6.0	100.0	4.2
1EYHA	87.7	100.0	3.5	69.0	87.5	5.3	100.0	3.5
1FQIA	82.2	88.9	1.6	74.8	88.9	4.6	88.9	1.5
1GAKA	87.4	100.0	7.8	68.0	83.3	6.6	100.0	4.5
1GYUA	86.8	100.0	1.1	75.4	100.0	2.1	100.0	0.9
1IAPA	82.1	100.0	6.1	78.8	100.0	5.7	100.0	5.4
1ICXA	84.8	100.0	1.7	76.1	90.0	2.1	100.0	1.6
1J27A	96.2	100.0	0.5	71.3	83.3	2.4	100.0	0.5
1JL1A	75.5	100.0	4.1	66.7	100.0	5.4	100.0	4.0
1LKIA	75.9	80.0	10.3	44.1	80.0	16.8	80.0	10.3
1LMIA	53.7	66.7	2.8	42.5	66.7	3.8	66.7	2.5
1OXJA	83.5	100.0	6.3	76.2	100.0	4.6	100.0	2.3
1OZ9A	91.1	100.0	1.0	79.3	88.9	2.0	100.0	0.8
1PBVA	93.9	100.0	0.8	89.3	100.0	1.4	100.0	0.6
1PKOA	77.1	100.0	1.8	62.8	88.9	2.6	100.0	1.6
1Q5ZA	76.3	100.0	3.2	64.0	100.0	3.8	100.0	1.3
1RJ1A	90.2	100.0	6.0	86.6	100.0	7.4	100.0	5.3
1T3YA	73.0	100.0	2.7	77.8	100.0	2.2	100.0	2.0
1TP6A	75.5	88.9	2.6	58.8	88.9	5.0	88.9	2.6
1TQGA	96.6	100.0	0.8	82.2	100.0	4.0	100.0	0.5
1TZVA	84.8	100.0	6.4	80.0	100.0	7.0	100.0	6.1
1UAIA	68.3	93.3	1.9	64.8	86.7	2.0	100.0	1.5
1ULRA	90.2	100.0	0.9	76.5	100.0	2.3	100.0	0.7
1VINA	83.5	100.0	2.1	71.8	83.3	4.2	100.0	1.8
1X91A	88.6	100.0	2.6	79.2	80.0	4.3	100.0	2.6
1XAKA	51.2	83.3	2.8	27.3	50.0	2.3	83.3	2.8
1XKRA	85.0	92.9	1.5	80.4	85.7	1.2	92.9	1.2
1XQOA	71.8	92.9	4.5	62.5	85.7	4.3	92.9	3.3
1Z3XA	82.6	92.3	1.3	70.6	84.6	7.9	100.0	3.8
2AP3A	81.6	100.0	6.4	76.3	100.0	12.0	100.0	6.0
2BK8A	94.0	100.0	0.4	72.9	100.0	1.9	100.0	0.4
2CWRA	77.4	100.0	2.3	75.8	87.5	1.3	100.0	1.6
2EJXA	71.4	90.0	2.7	47.3	70.0	6.6	90.0	2.7
2F1SA	83.3	91.7	1.5	76.0	83.3	2.1	91.7	1.3
2FC3A	84.4	100.0	1.6	68.7	88.9	3.8	100.0	1.4
2FM9A	85.5	100.0	5.7	85.2	100.0	2.8	100.0	2.6
2FRGP	69.1	88.9	2.1	68.8	88.9	2.5	88.9	2.0
2GKGA	90.0	100.0	0.8	73.6	80.0	1.3	100.0	0.7
2HUJA	94.0	100.0	1.5	83.8	100.0	5.3	100.0	1.5
2IU1A	82.0	90.0	2.7	77.7	90.0	11.4	90.0	2.6
2JLIA	65.2	100.0	3.0	64.4	100.0	4.3	100.0	3.0
2LISA	88.2	100.0	4.6	73.1	100.0	7.4	100.0	4.6
2OF3A	85.4	100.0	7.9	78.2	87.5	5.3	100.0	5.5
2OSAA	79.4	88.9	2.4	70.3	88.9	4.0	88.9	2.1
2QZQA	48.2	85.7	3.8	43.3	85.7	4.5	85.7	3.5
2R0SA	68.6	84.6	2.2	61.3	69.2	3.1	84.6	2.2
2RB8A	80.8	100.0	1.4	82.4	100.0	1.3	100.0	1.0
2RCIA	60.7	90.0	5.3	52.8	90.0	6.3	90.0	4.4
2V75A	76.9	100.0	3.6	72.6	100.0	4.0	100.0	3.2
2VQ4A	74.2	100.0	2.1	71.0	75.0	1.3	100.0	2.0
2WJ5A	83.7	100.0	0.7	73.5	100.0	1.7	100.0	0.8
2WWEA	79.8	100.0	4.3	66.7	71.4	3.2	100.0	4.1
2YV8A	81.2	91.7	1.0	75.3	83.3	1.3	91.7	0.5
2YXFA	69.2	100.0	2.3	55.7	100.0	3.4	100.0	2.1
2YYOA	69.1	100.0	2.2	62.0	100.0	3.0	100.0	2.1
2ZCOA	83.8	93.3	5.7	81.0	100.0	8.3	100.0	5.1
3B5OA	72.3	100.0	9.1	58.6	88.9	8.1	100.0	7.4
3CTGA	83.1	100.0	1.4	67.5	77.8	2.4	100.0	1.4
3CX2A	75.4	100.0	1.3	67.2	100.0	2.4	100.0	1.4
3FH2A	96.0	100.0	0.4	89.4	100.0	3.4	100.0	0.3
3FHFA	68.9	91.7	5.4	63.3	91.7	8.4	100.0	6.7
3FRRA	93.0	88.9	5.4	86.2	88.9	6.5	88.9	5.3
3HVWA	60.0	90.9	3.9	54.6	72.7	4.0	90.9	2.5
3IV4A	83.1	100.0	1.7	81.0	100.0	2.0	100.0	1.3
3NE3B	80.9	100.0	1.3	76.7	100.0	2.3	100.0	1.1
3OIZA	68.0	100.0	3.6	64.5	100.0	3.9	100.0	3.3
***avg***	79.6	96.1	3.1	70.2	90.3	4.3	96.6	2.7
***stdev***	10.6	6.4	2.2	11.9	10.7	2.8	6.4	1.9

The table depicts pool Q3 score, %found (percent of native SSEs identified by predictions) and average shifts for the pools generated using secondary structure prediction methods PSIPRED and JUFO for all of the 66 proteins in the benchmark set. The last two rows show the average and the standard deviation for pool agreement score and Q3 measure. The statistics is repeated for the combined pool of PSIPRED and JUFO.

### Two-stage Assembly and Refinement Protocol Separates Moves by type and Amplitude

BCL::Fold samples the conformational search space by a variety of SSE-based moves. These moves coupled with exclusion of loop residues, provide a significant advantage in fast sampling of different topologies. The minimization process is divided into two stages. The “assembly” stage consists of large amplitude translation or rotations and moves that add or remove SSEs (Movies in Table S5: row “Assembly). Other moves central to this phase shuffle β-strands within β-sheets or break large β-sheets to create β-sandwiches. The “refinement” stage focuses on small amplitude moves that maintain the current topology but optimize interactions between SSEs and introduce bends into SSEs (Movies in Table S5: row “Refinement). Currently both stages utilize the same energy function (compare companion BCL::Score manuscript).

Once the SSE pool is input, the algorithm initializes the energy functions and move sets with corresponding weight sets for assembly and refinement stages. A starting model for the minimization is created by inserting a randomly selected SSE from the pool into an empty model. The starting model is passed to the minimizer which executes assembly and refinement minimization. The assembly stage terminates after 5000 steps in total or after 1000 consecutive steps that did not improve the score. The refinement stage terminates after 2000 steps in total or 400 consecutive steps that did not improve the score. In general, a move can result in one of four outcomes (Figure S1): “improved” in score, “accepted” through Metropolis criterion, “rejected” as score worsened, or “skipped” as SSE elements required for the move are not present in the model. The temperature is adjusted dynamically based on the ratio of accepted steps (see Methods). The Metropolis outcome “skipped” is introduced in the algorithm to deal with the non-applicability of certain steps to the model in the optimization. Although particular moves could be disabled before selecting them randomly, they are selected and counted as “skipped”.

**Figure 3:**
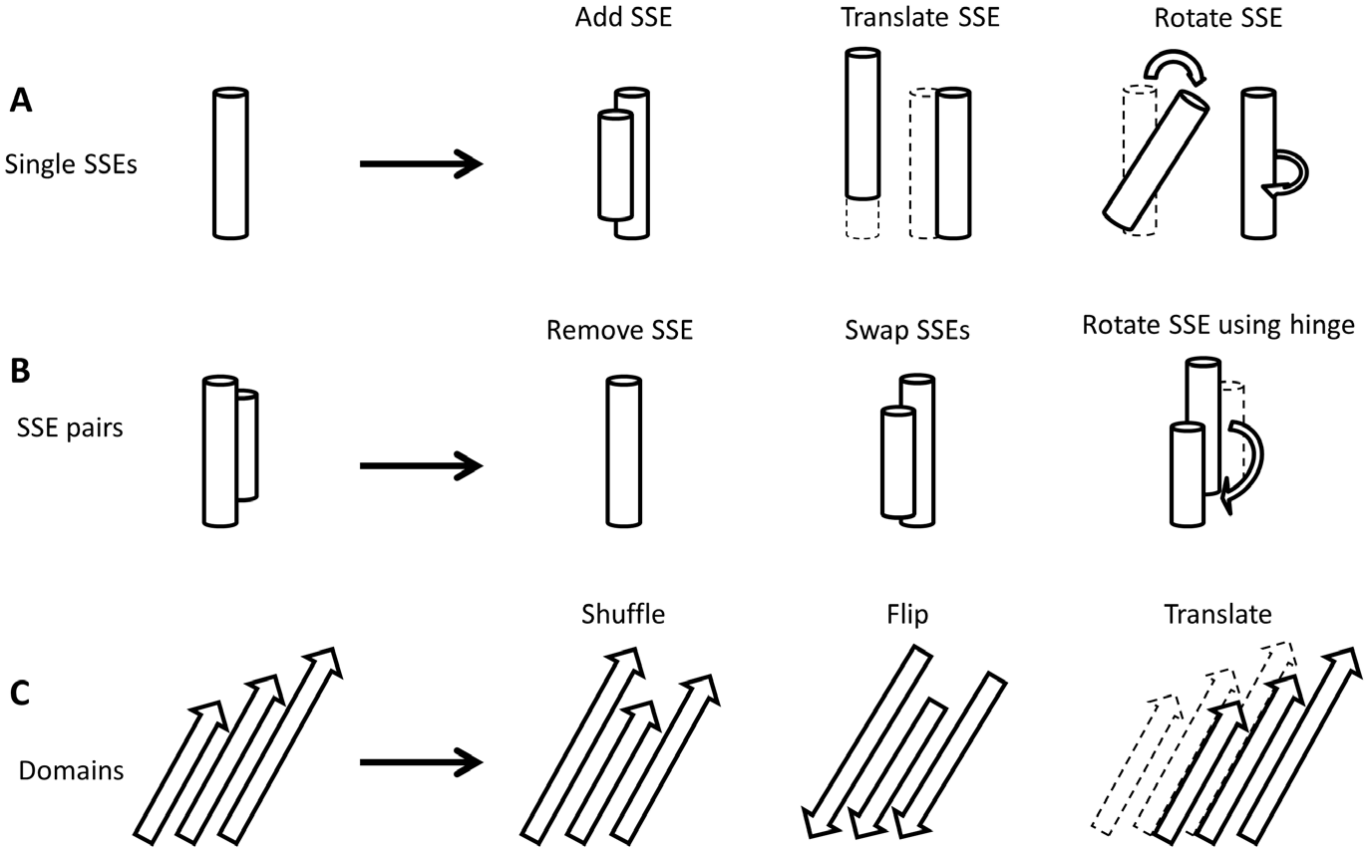
SSE-based moves allow rapid sampling in conformational search space. The types of moves used in the BCL::Fold protocol are explained with a representative move set. (**A**) Single SSE moves: These moves can include adding a new SSE to the model from the pool as well as translations/rotations/transformations. (**B**) SSE pair moves: One of the SSEs in the pair can be removed, the locations can be swapped and one can be rotated around the other SSE which is used as a hinge to define rotation axis. (**C**) Domain based moves: These moves act on a collection of SSEs such as a helical domain or β-sheet. The examples show how the locations of strands can be shuffled within a β-sheet or how an entire β-sheet can be flipped or translated.

A comprehensive list of all moves used in BCL::Fold is given in Table S1 (assembly stage) and Table S2 (refinement) along with brief descriptions. The moves are categorized into six main categories; (1) adding SSEs, (2) removing SSEs, (3) swapping SSEs, (4) single SSE moves, (5) SSE-pair moves, and (6) moving domains, i.e. larger sets of SSEs. Representations for a selection of moves used in BCL::Fold are illustrated in Figure 3. SSE, SSE-pair and domain moves are further categorized into specific versions for α-helices, and β-strands or α-helix domains, and β-sheets, resulting in a total of nine individual categories. The relative probability or weight for each move category is initialized at the beginning of the minimization and depends on the SSE content of the pool. For example, β-sheet moves are excluded if the given pool contains only α-helices. This procedure limits the number of move trials that are unsuccessful or “skipped” because the needed SSEs are not in the model. As mentioned in the previous section, depending on the amplitude, moves are categorized to be used in either the assembly stage or the refinement stage. Out of 107 moves, 72 are used exclusively in assembly and 33 are used exclusively in refinement. Resizing SSEs (“sse_resize_nterm” and “sse_resize_cterm”) are the only moves used in both stages. Table S1 and Table S2 also provides statistics of how frequently each move leads to an improved, accepted, rejected, or skipped status as well as the average improvement in the score observed for all the improved steps based on statistics collected on the 66 benchmark proteins. Assembly moves have an average score improvement of −100±78 BCLEU (Table S1) while the refinement moves have an average score change of −15±10 BCLEU (Table S2).

The five individual moves with largest score improvements either add SSEs or manipulate β-strands, including “add_strand_next_to_sheet”, “sheet_pair_strands”, “add_sse_short_loop” and “add_sse_next_to_sse”. At the same time, these moves also lead to improved models with a relatively high percentage, ranging from 10% to 30% of the cases where the move is not skipped. On the other hand, these moves, especially ones including adding SSEs, also lead to a high percentage of skipped steps. This is due to the fact that the weight for these moves is currently not dynamically adjusted depending on how many SSEs are already added to the model. On the contrary, moves with small average score improvements are less frequently skipped but also less frequently accepted.

It is somewhat misleading to analyze the moves in isolation as rearranging or refining the topology often requires a series of different moves and success of one move relies on the availability of suitable companion moves. For further investigation of which types of moves are more complementary to each other, trajectories from runs for all benchmark proteins were analyzed. For each pair of move type 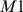 and 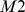, a non-symmetric correlation measure 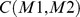 was calculated using the equation below which measures how likely it is that a move 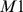 leads to a step where 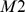 is applied to improve the current model.
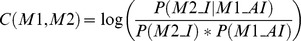
where 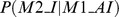 is the fraction of 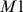 improved moves preceded by a 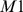 accepted or improved move in the previous 50 steps; 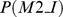 is the fraction of 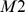 moves that lead to an improved step; and 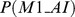 is the fraction of 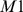 moves that lead to an improved or accepted step. If any of the probabilities are 0, the correlation value is assigned as -3. A higher correlation value 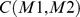 indicates that move 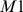 is more likely to be observed as an improved or rejected step in close proximity before a 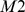 move is observed in an improved step.

**Figure 4:**
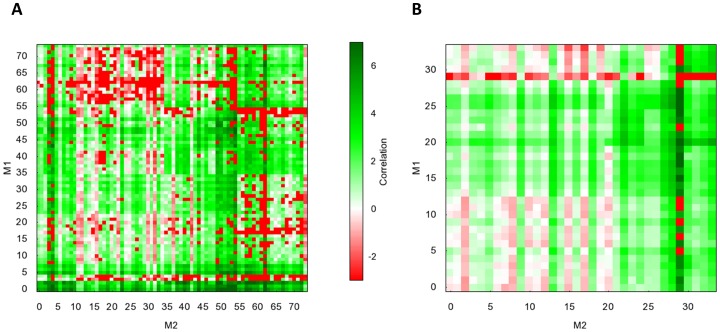
Correlation of moves used in BCL::Fold. The correlation of all move pairs is depicted as heat maps for (**A**) assembly moves (**B**) refinement moves. For both heat maps, the moves are ordered in the same order as in Tables S1 and S2 respectively.

Figure 4 depicts heat maps for pairwise correlation values for all assembly moves (Figure 4A) and refinement moves (Figure 4B). As observed in both panels, certain move pairs have an increased chance of producing energetically favorable models. As seen in the heat map for assembly (Figure 4A), add moves (#1–3) often precede successful move combinations since whenever a new SSE is added, it is expected that is not at the energetically optimal placement and will need further moves to optimize the location of this SSE. A similar pattern can also be observed for swap moves (#6–8). On the other hand, α-helix domain moves (#50–55) have a tendency to be preceded by individual SSE (#9–24) or α-helix moves (#25–35), while β-sheet moves (#56–74) are more frequently preceded by individual β-strand moves (#36–43). The refinement moves (Figure 4B) have a less pronounced pattern compared to assembly moves since they introduce smaller changes. Less often a second move is needed to compensate for an accepted first move. α-helix pair moves (#26–27) preferably precede α-helix domain moves (#28–30), while the “strand_translate_z_small” move (#21) is followed by β-sheet moves (#31–35).

### BCL::Fold Samples Native-like Topologies for 92% of Benchmark Proteins

**Figure 5:**
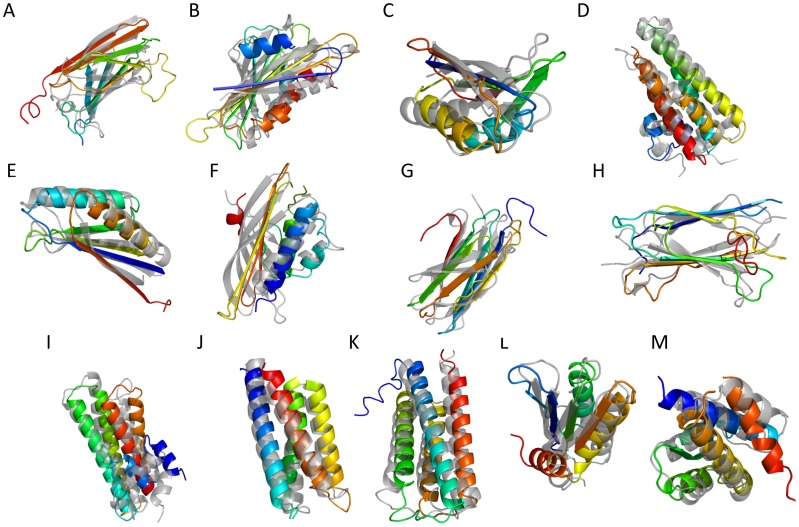
Structures for a selection of best RMSD100 complete models generated by BCL::Fold. Best complete models by RMSD100 with a predicted pool generated by BCL::Fold for a selection of proteins. The generated models are rainbow colored and superimposed with the native structure (gray) for the following proteins. The numbers refer to the RMSD100 of the models: (**A**) 1GYUA –6.39Å (**B**) 1ICXA –6.46Å (**C**) 1ULRA –4.73Å (**D**) 1X91A –4.49Å (**E**) 1J27A –3.72Å (**F**) 1TP6A –6.83Å (**G**) 2CWRA −7.61Å (**H**) 2RB8A –5.09Å (**I**) 1RJ1A –5.33Å (**J**) 1TQGA 2.44Å (**K**) 2HUJA –3.37Å (**L**) 3OIZA –6.21Å (**M**) 2V75A –3.55Å.

**Figure 6:**
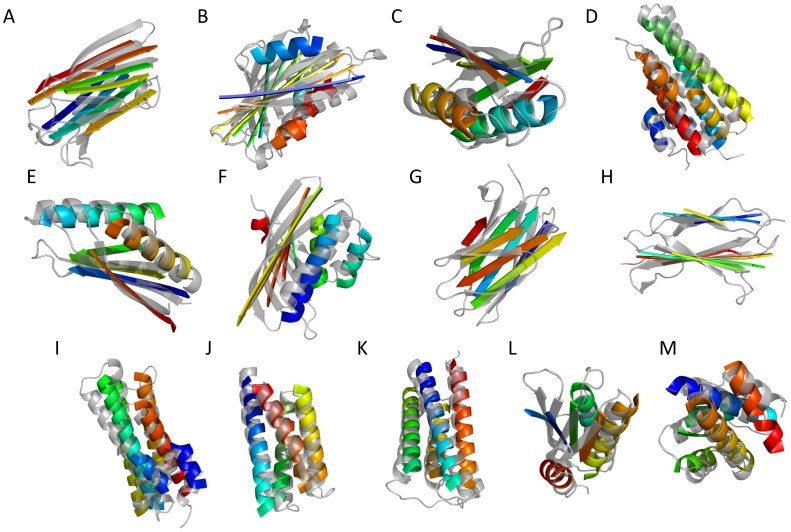
Structures for a selection of best RMSD100 SSE-only models generated by BCL::Fold. Best SSE-only models by RMSD100 with a predicted pool generated by BCL::Fold for a selection of proteins. The generated models are rainbow colored and superimposed with the native structure (gray) for following proteins. The numbers refer to the RMSD100 of the models: (**A**) 1GYUA –4.11Å (**B**) 1ICXA –6.07Å (**C**) 1ULRA –3.61Å (**D**) 1X91A –4.08Å (**E**) 1J27A –3.15Å (**F**) 1TP6A –5.74Å (**G**) 2CWRA −5.66 Å (**H**) 2RB8A –2.91Å (**I**) 1RJ1A –4.86Å (**J**) 1TQGA –1.92Å (**K**) 2HUJA –2.57Å (**L**) 3OIZA –5.76Å (**M**) 2V75A –3.11Å.

10,000 structural models were generated for each protein in the benchmark set using BCL::Fold. Two separate runs were performed with BCL::Fold, one using a SSE pool composed of native SSE definitions as computed from the experimental structures using DSSP [73]. A second run was performed using a BCL::SSE predicted pool. To facilitate the analysis of models, loops were constructed using a rapid CCD based method [38] (see Methods and Movies in Table S5: rows “Loop grow”, “Loop close” and “Loop force close”). However, in the present analysis we focus on placement of SSEs to form the topology and evaluate models using two qualities measures; RMSD100 (C_α_ root mean square deviation normalized to a protein length of 100 residues [74]) and Contact Recovery (CR). CR measures percentage of native contacts recovered, where a contact is defined as the presence of two amino acids of at least 12 residues sequence separation and<8Å Cβ distance. The average and standard deviations of RMSD100 and CR values of the best models generated by these runs can be found in Table 3 **.** Figures 5 and 6 illustrate the best RMSD100 SSE-only and complete structural models generated by BCL using predicted SSE pools for a selection of benchmark proteins.

**Table 3:** Best RMSD100 and CR values for models generated by BCL and Rosetta.

	RMSD100 [Å]					cr12 [%]				
pdb id	BCL_N-SSE_	BCL_N_	BCL_P-SSE_	BCL_P_	Rosetta	BCL_N-SSE_	BCL_N_	BCL_P-SSE_	BCL_P_	Rosetta
1BGCA	2.94	4.25	5.41	6.29	7.06	61.11	59.26	45.37	49.07	42.59
1EYHA	6.06	6.92	5.87	7.20	4.30	28.69	31.15	41.80	37.70	40.77
1FQIA	7.17	7.60	6.20	8.06	5.37	38.46	36.92	40.00	44.62	32.58
1GAKA	4.90	7.06	6.38	7.69	4.60	42.59	42.59	29.63	32.41	66.67
1GYUA	4.41	6.39	4.11	6.39	5.96	58.68	58.68	61.16	61.16	51.24
1IAPA	6.46	7.55	7.38	8.23	5.65	27.12	27.12	22.03	22.03	19.11
1ICXA	6.51	6.80	6.07	6.46	5.59	45.74	46.28	51.06	48.40	37.44
1J27A	3.20	3.62	3.15	3.72	4.49	71.20	70.40	68.80	70.40	54.07
1JL1A	6.04	8.01	6.75	8.19	8.26	39.05	43.33	31.43	34.76	26.52
1LKIA	2.90	5.40	7.07	9.10	7.18	59.29	59.29	23.01	29.20	36.70
1LMIA	5.82	8.60	6.72	9.97	9.49	49.65	48.94	29.08	31.21	26.95
1OXJA	6.42	7.67	7.21	7.79	6.75	44.68	45.74	30.85	30.85	45.45
1OZ9A	5.78	6.88	5.22	5.93	5.39	38.36	43.40	40.25	40.88	37.50
1PBVA	8.02	9.02	8.75	9.14	6.47	28.30	28.30	30.19	31.13	40.00
1PKOA	6.03	7.81	7.58	8.15	8.43	43.07	40.88	38.69	39.42	27.74
1Q5ZA	5.64	8.03	7.28	8.56	8.93	40.00	40.00	41.54	43.08	21.28
1RJ1A	4.66	5.52	4.86	5.33	3.33	48.43	48.43	55.97	57.23	45.56
1T3YA	5.71	5.92	5.86	6.56	6.27	49.61	51.94	42.64	44.96	34.06
1TP6A	4.91	5.65	5.74	6.83	5.25	49.65	53.15	46.15	47.55	44.59
1TQGA	1.91	2.19	1.92	2.44	1.41	76.40	76.40	76.40	76.40	93.55
1TZVA	4.55	6.01	4.89	5.58	3.20	46.28	46.28	39.67	39.67	45.45
1UAIA	6.13	7.95	7.24	9.05	9.62	38.58	37.01	29.53	31.50	16.54
1ULRA	3.17	4.82	3.61	4.73	4.18	62.14	63.11	66.02	70.87	53.98
1VINA	7.42	8.53	7.62	8.68	8.48	25.46	25.46	20.83	21.76	21.98
1X91A	2.40	3.30	4.08	4.49	2.49	77.64	77.64	48.45	49.07	68.02
1XAKA	8.17	9.53	5.28	7.77	8.67	53.03	53.03	31.82	48.48	34.85
1XKRA	6.14	7.29	8.04	8.47	8.79	28.47	30.29	27.37	32.48	24.51
1XQOA	8.05	8.71	7.50	8.20	9.16	18.82	18.28	18.82	19.35	14.73
1Z3XA	7.74	9.19	7.58	9.59	8.44	24.27	24.27	22.33	22.33	30.37
2AP3A	2.78	3.16	3.67	6.06	4.17	52.52	51.08	43.17	42.45	52.26
2BK8A	5.09	6.89	4.81	7.13	4.27	56.41	57.69	55.13	55.13	80.77
2CWRA	5.99	6.05	5.66	7.63	7.46	45.80	45.04	44.27	48.85	26.72
2EJXA	6.28	6.64	7.48	7.89	5.17	41.43	42.14	29.29	35.00	38.82
2F1SA	6.68	7.93	7.61	8.26	7.34	27.27	28.28	25.25	26.26	27.27
2FC3A	4.90	7.39	5.63	6.78	5.75	34.62	40.00	36.92	46.92	25.36
2FM9A	6.22	7.05	6.26	6.95	6.37	24.05	24.05	21.52	22.78	25.14
2FRGP	4.67	5.69	5.38	6.41	6.53	57.02	56.14	53.51	53.51	35.96
2GKGA	3.43	4.31	3.89	4.40	3.45	52.10	52.94	47.06	48.74	58.20
2HUJA	2.12	2.98	2.57	3.37	3.65	71.31	71.31	66.39	67.21	47.29
2IU1A	6.70	7.99	6.84	8.04	7.16	25.16	25.79	20.13	23.27	27.06
2JLIA	6.18	7.11	6.93	8.14	6.46	46.59	46.59	37.50	42.05	36.89
2LISA	4.77	6.03	5.47	7.22	5.71	43.33	43.33	41.11	45.56	59.79
2OF3A	8.92	9.26	8.25	9.30	8.30	20.24	20.65	19.43	20.65	26.58
2OSAA	6.55	7.78	7.21	8.82	8.05	27.14	27.14	28.57	32.86	24.86
2QZQA	5.48	8.73	6.10	8.40	9.89	63.24	61.03	47.06	52.94	34.81
2R0SA	6.95	9.53	7.80	10.29	10.27	24.49	24.49	23.13	23.13	21.30
2RB8A	3.27	5.07	2.91	5.09	4.91	62.11	61.05	68.42	70.53	60.00
2RCIA	5.32	6.99	9.17	10.20	10.29	47.50	48.75	22.08	24.58	16.06
2V75A	3.26	3.66	3.11	3.55	2.29	67.12	65.75	60.27	60.27	85.71
2VQ4A	4.18	6.65	5.28	7.20	9.18	57.94	57.01	59.81	59.81	36.45
2WJ5A	5.80	8.48	6.41	8.63	7.86	67.80	66.10	71.19	71.19	77.97
2WWEA	4.92	6.43	5.30	6.22	5.97	45.95	48.65	48.65	48.65	41.38
2YV8A	5.71	7.85	5.54	7.48	8.53	47.62	47.02	43.45	41.07	26.19
2YXFA	5.84	7.28	6.13	6.65	4.38	51.46	51.46	48.54	51.46	53.40
2YYOA	6.68	8.55	6.72	7.94	9.13	37.91	39.22	40.52	41.18	23.53
2ZCOA	7.75	8.42	7.63	8.33	8.20	18.28	19.03	18.28	19.03	17.18
3B5OA	6.46	7.28	8.62	8.96	9.10	23.11	23.11	9.78	15.11	11.43
3CTGA	5.52	6.89	5.61	6.93	4.07	52.11	53.52	45.07	50.70	48.72
3CX2A	4.96	7.88	7.27	7.04	8.20	54.37	54.37	49.51	54.37	47.57
3FH2A	6.37	7.34	6.35	7.55	4.73	33.33	33.97	27.56	28.21	41.85
3FHFA	8.60	9.17	7.81	8.88	7.54	19.42	21.36	16.50	18.45	27.07
3FRRA	6.50	7.50	4.53	5.87	5.46	30.82	30.82	33.33	34.59	45.25
3HVWA	6.10	8.02	6.48	6.49	6.69	39.61	40.26	30.52	37.66	25.95
3IV4A	3.34	4.68	4.54	5.80	3.98	60.61	61.62	52.53	49.49	40.95
3NE3B	5.01	5.65	6.41	7.01	5.60	45.45	48.25	48.25	51.05	34.10
3OIZA	4.48	5.00	5.76	6.21	4.20	50.00	50.96	30.77	41.35	56.76
**avg**	**5.50**	**6.81**	**6.04**	**7.21**	**6.42**	**44.55**	**44.96**	**39.63**	**41.88**	**39.42**
**stdev**	**1.61**	**1.73**	**1.58**	**1.68**	**2.16**	**15.13**	**14.81**	**15.31**	**14.96**	**17.52**

The table lists for all proteins, the best RMSD100 and best CR observed for models generated by BCL and Rosetta. BCL results are presented in 4 columns: SSE-only models using native SSE definitions (BCL_N-SSE_), complete models using native SSE definitions (BCL_N_), SSE-only models using predicted SSE definitions (BCL_P-SSE_), complete models using predicted SSE definitions (BCL_P_). The 5th columns under RMSD100 and CR are for Rosetta models.

BCL::Fold using the correct secondary structure achieved RMSD100-values of 5.5±1.6Å (SSE only models) and 6.8±1.7Å (complete models). For simulations with predicted SSEs, RMSD100 values of 6.0±1.6Å (SSE only models) and 7.2±1.7Å (complete models) were obtained. For comparison, Rosetta [28] generated models with RMSD100-values of 6.4±2.1Å. BCL::Fold improved the RMSD100 when compared with Rosetta in 24 cases (36%) with correct SSE definitions and in 19 cases (29%) using a predicted SSE pool. When CR values are considered, BCL::Fold using the correct secondary structure achieved 44.6±15.1 (SSE only models) and 45.0±15.0 (complete models). For simulations with predicted SSEs, CR-values of 39.6±15.3 (SSE only models) and 41.9±15.0 (completed models) were obtained. For comparison, Rosetta generated models with CR-values of 39.4±17.5. BCL::Fold improved the recovery of native contacts when compared with Rosetta in 47 cases (71%) with correct SSE definitions and in 40 cases (60%) using a predicted SSE pool.

When best models by RMSD100 are considered, BCL::Fold was able to predict the correct topology in 61 cases (92%) independent of usage of correct or predicted SSE pools. Models with correct topology, or native-like models, were defined as having an RMSD100 value of less than 8.0Å. After loop construction, native-like models are obtained for 50 cases (75%) using correct SSE predictions and 41 cases (62%) using a predicted SSE pool. In comparison, Rosetta constructed native-like models for 45 cases (68%). When a CR value of>20% is taken as cutoff, success rates change to 64 cases (97%) and 62 cases (94%), respectively, for BCL::Fold and to 60 cases (91%) for Rosetta. We attribute the deterioration of BCL::Fold models after loop construction mostly to limited sampling performed at this stage of the protocol as the present work focuses on topology assembly.

For further analysis, the best-scoring 100 models (1%) for each protein and each method were kept. For these subsets the percentage of targets where the best model by RMSD100 was below 8.0Å were calculated. BCL::Fold using correct SSEs was able to generate a<8.0Å RMSD100 model in top 1% by score for 56% of targets (SSE only models) and 39% (complete models). These values for BCL::Fold simulations with predicted pools were 44% (SSE only models) and 22% (complete models), while being 43% for Rosetta. This was followed by a similar analysis where CR measure was used instead of RMSD100. BCL::Fold using correct SSEs was able to generate>20% CR models for 74% of targets (SSE only models) and 79% (complete models). For simulations with predicted pools, CR values were 73% (SSE only models) and 76% (complete models), while being 74% for Rosetta.

**Figure 7:**
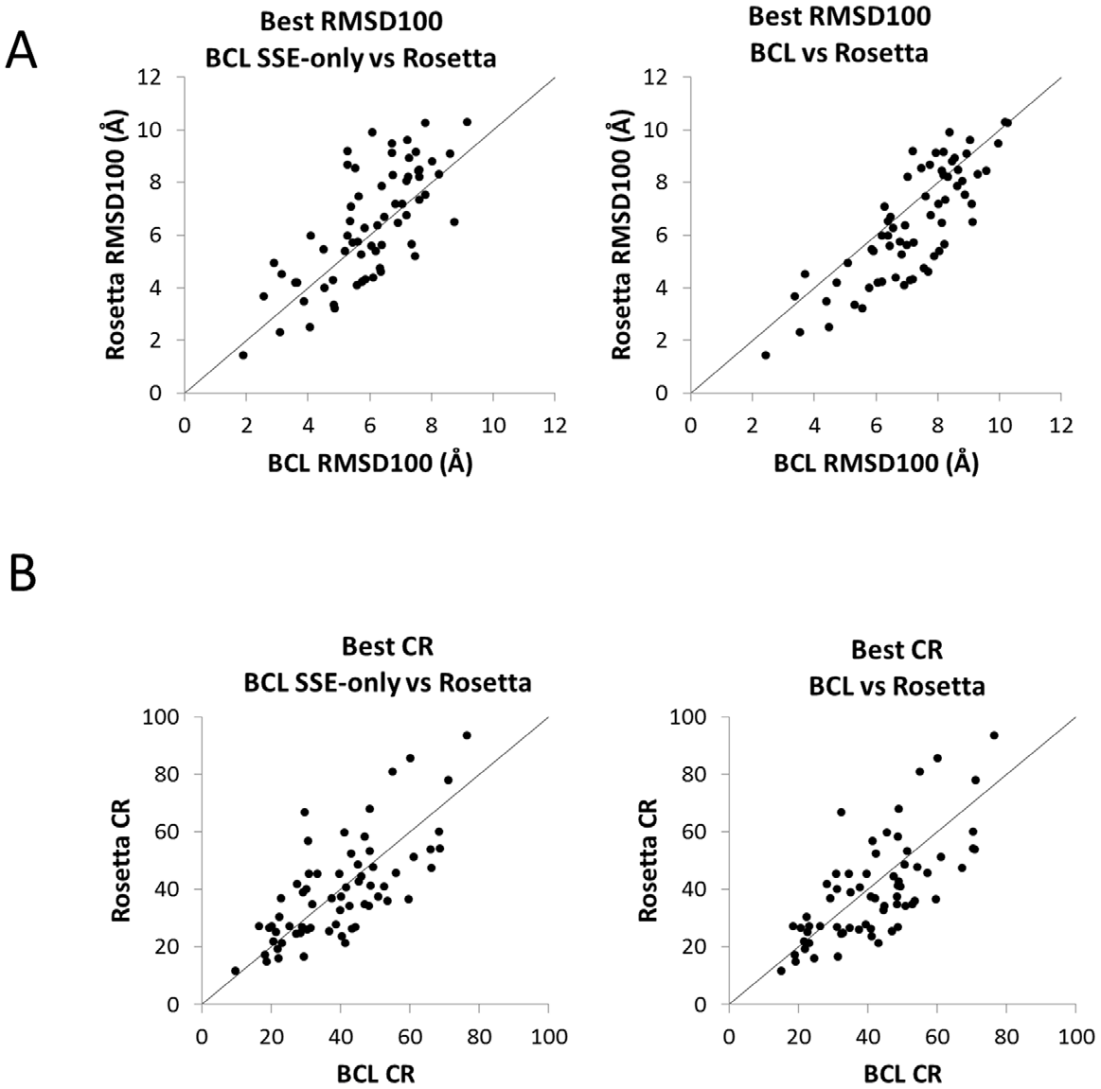
Comparison of best RMSD100 and CR values for BCL and Rosetta. Scatter plot comparing (**A**) best RMSD100 or (**B**) best CR SSE-only (left) and complete (right) BCL models vs. Rosetta models. The BCL models considered are from BCL::Fold runs using predicted SSE pools. (**B**) Scatter plot comparing best CR SSE-only (left) and complete (right) BCL models vs. Rosetta models. The BCL models considered are from BCL::Fold runs using predicted SSE pools.

Figure 7 provides the comparison of best RMSD100 and CR values achieved for all benchmark proteins between Rosetta and BCL. When RMSD100 values are considered, SSE-only models for BCL runs with predicted SSE pools (Figure 7A left panel) provide a better performance than Rosetta. As explained earlier, SSE-only models are given an advantage due to the smaller number of atoms over which RMSD100 values are calculated for these models owing to the lack of flexible loop regions. When complete models are compared with Rosetta (Figure 7A right panel); it is observed that Rosetta produces lower RMSD100 models for more targets although performance correlates very well. Figure 7B displays CR values giving a slight advantage to the BCL in recovering native-like SSE contacts. These results are promising for BCL::Fold especially given the fact that BCL::Fold was designed with a focus on getting the SSE topology correct.

**Figure 8:**
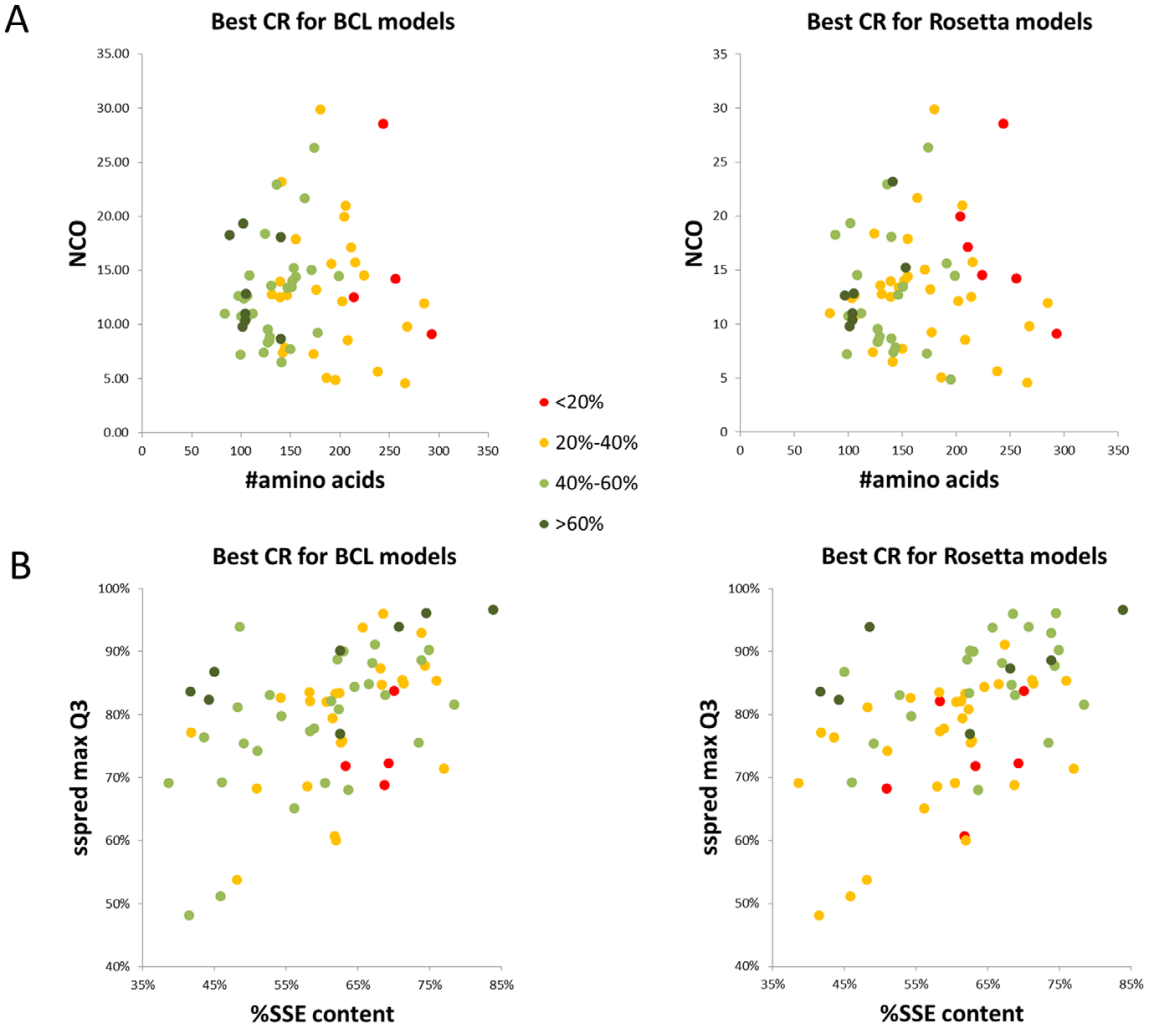
Determinants of high CR values in BCL and Rosetta models. (**A**) Plot of sequence length vs. relative contact order (RCO) for all benchmark proteins. (**B**) Plot of percentage of amino acids found in SSEs vs. maximum Q3 value achieved from JUFO or PSIPRED pools for all benchmark proteins. Individual plots are presented for models from BCL::Fold runs using predicted SSE pools (left panels) and Rosetta models (right panels). Points in both (A) and (B) are colored according to the best CR value achieved for that benchmark protein in BCL runs using predicted SSE pools and complete models;<20% (red), 20% to 40% (orange), 40% to 60% (green) and>60% (dark green).

BCL::Fold performance varies between different targets, as observed in the plots mentioned above. We wanted to investigate whether there is a correlation of performance with sequence length, fold complexity, secondary structure content, or accuracy of the secondary structure prediction. For this purpose, for each benchmark protein, the sequence length is plotted against NCO values (Figure 8A left panel) and each point is colored according to the highest CR value achieved for the complete models generated by BCL::Fold runs using predicted SSE pools for that protein. As seen in the plots, the best performing proteins (>60%), are limited to<150 residue proteins. On the other hand, 40% to 60% CR values were achieved for proteins up to 200 residues, and 20%–40% CR values were attainable for proteins up to 275 residues. Similar numbers were observed for Rosetta models (Figure 8A right panel).

### Accurate Secondary Structure Improves Quality of BCL::Fold Models only Slightly

Comparison of BCL::Fold runs with either predicted or correct SSEs (Table 3) reveals that using native SSE definitions provides an average improvement of 0.64Å in RMSD100 for SSE only models and 0.40 Å RMSD100 for complete models after loop construction. Although the effect of secondary structure prediction accuracy on average of best RMSD100 models is modest, this effect is not directly related to Q3 values, but rather due to the nature of the BCL::Fold assembly protocol. One interesting example is 1LKIA, a 180 residue protein with Q3 values of 75.9 (PSIPRED) and 44.1 (JUFO). Although this protein has a mid-range PSIPRED Q3 value, it exhibits the largest deterioration in both RMSD100 and CR, which is more likely to be explained by the high average shift values; 10.3 residues (PSIPRED) and 16.8 residues (JUFO). Another such example is 1LMIA, which has low Q3 value of 53.7 and 42.5 for PSIPRED and JUFO respectively, accompanied by a low rate correct SSE identification of 67%. On the other hand, if the secondary structure prediction is extremely accurate as in the case of 1TQGA, 1J27A, 3FH2A, 2BK8A (all with PSIPRED Q3>94.0), RMSD100 values deteriorate less than 0.3Å when moving from perfect to predicted secondary structure. Although accurate secondary structure prediction improves the overall accuracy of BCL::Fold, the results indicate that it is not a requirement. As described in Table S1 and Table S2, BCL::Fold utilizes a set of moves to dynamically resize and split SSEs during the minimization to compensate for the inaccuracies in secondary structure prediction.

The SSE content (percentage of residues in a sequence that reside in an SSE as opposed to coil) versus maximum Q3 value of the pool generated (using the highest Q3 value of the PSIPRED and JUFO predictions) for each benchmark protein is plotted in Figure 8B. Each point is colored according to the best CR value achieved for that target in complete models generated in BCL::Fold runs using predicted SSE pools (Figure7B left panel). Nearly all targets with highest CR values (>60% colored purple) have ∼80% or higher Q3 values, although the SSE content for these targets can range from as little as 40% to as high as 85%. Similar plots are also provided for Rosetta models for comparison (Figure 8B right panel). When models for the proteins with<75% maximum Q3 values are considered, a clear decline in the best CR values could be observed for Rosetta models in comparison to BCL models. For these proteins with relatively low confidence secondary structure predictions, the Rosetta generated best CR model was<20% for 4 cases, and between 20% and 40% for 11 cases, while these numbers were 3 and 6 respectively for BCL::Fold.

### BCL::Fold BETA was Evaluated in CASP9 Experiment

All techniques for protein structure prediction are evaluated every two years via the Critical Assessment of Techniques for Protein Structure Prediction (CASP) experiment [75], [76], [77]. An early version of BCL::Fold (BCL::Fold BETA) participated in CASP9 and predictions were submitted for 58 of 63 targets given in the human predictor category. For each target 50,000 models were generated, the top 10,000 by BCL score were selected for clustering analysis. The five best scoring models as well as the best scoring models in each of the large clusters (∼20) underwent loop construction and side chain packing using Rosetta. The five models for submission were selected from these full atom models as the largest cluster centers. In cases were a template was readily available, the fifth model for submission was the BCL::Fold model with the smallest RMSD to the comparative model built by MODELLER [78]. This approach was chosen to test the BCL::Fold sampling independent from BCL::Score (compare companion BCL::Score manuscript).

Targets in CASP9 were biased towards proteins of known fold. In fact, only 14 out of the 60 human targets had no sequence detectable templates [79]. However, BCL::Fold treated all targets “free modeling (FM)” to maximally leverage the blind CASP experiment to test the algorithm. In cases where a template was available we would not expect to perform better than template-based methods. The remaining few cases represent a too small sample size to comprehensively compare BCL::Fold with other *de novo* protein structure prediction methods, also because of the BETA stage of that version. Therefore we present anecdotal examples where the potential of this early version of the algorithm became apparent. A more detailed evaluation will be performed during CASP10 in summer 2012.

**Figure 9:**
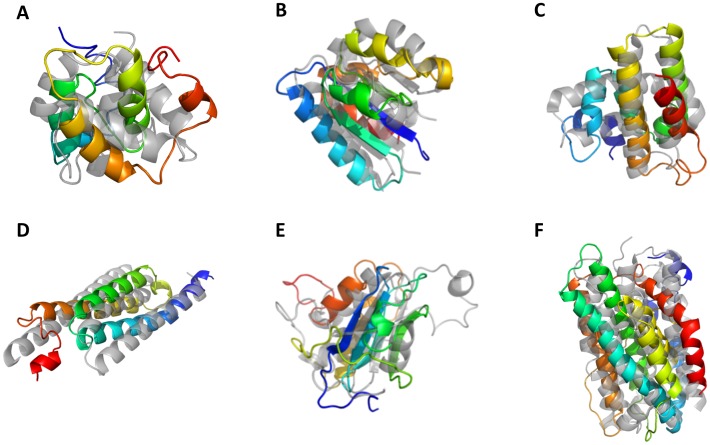
BCL::Fold results from CASP9. The best submitted model out of the 5 top submissions by RMSD (rainbow colored) superimposed with the native structure for (**A**) T0608_1–89 residues, 4.3Å RMSD (**B**) T0580 - 105 residues 4.44Å RMSD, (**C**) T0619 - 111 residues, 5.86Å RMSD (**D**) T0602 - 123 residues, 7.75Å RMSD (**E**) T0630 - 132 residues, 8.42Å RMSD (**F**) T0627 - 261 residues, 8.90Å RMSD.

For FM target T0608_1, the first submission by BCL::Fold had an RMSD of 4.3Å and ranked 9^th^ out of 132 groups (Figure 9). BCL::Fold was also able to produce native-like models and pick them for submission for the following targets; T0580 (105 residues 4.4Å RMSD), T0619 (111 residues 5.9Å RMSD), T0602 (123 residues 7.7Å RMSD), T0630 (132 residues 8.4Å RMSD), T0627 (261 residues 8.9Å RMSD).

### Conclusion

In conclusion, we demonstrate that assembly of SSEs is a viable approach to predict the topology of a protein of unknown fold. BCL::Fold assembles the correct topology for about 3 out of 5 proteins with sequence lengths ranging from 88 residues to 293 residues and 4 to 15 SSEs. BCL::Fold assembly runs range from 1 minute for the smallest protein to 10 minutes for the largest protein with a linear scaling (Figure S3). A detailed run-time comparison to other prediction methods was not conducted; however Rosetta and BCL::Fold per-model runtimes are currently at the same magnitude (Data for Rosetta not shown). With the maturation of the program code and the method, we expect that runtimes will decrease significantly, and due to the rapid sampling of topologies typically fewer models need to be generated as for this study.

The impact of predicted versus correct secondary structure is small, demonstrating that BCL::Fold can efficiently compensate for inaccuracies in secondary structure prediction. As mentioned above, BCL::Fold currently focuses on topological sampling of SSEs neglecting backbone flexibility within individual SSEs. This leads to increased RMSD100 values especially in β-sheet proteins where despite correct topology, the curvature of β-sheet is not correctly reproduced. With development of more efficient SSE backbone bending strategies BCL::Fold can overcome this limitation.

As expected, BCL::Fold’s overall performance, in terms of both RMSD100 and CR, is more robust for smaller proteins. There is a linear dependency, more clearly seen with decreasing CR values, the larger the protein, the larger the conformational space to be sampled. Out of 31 α-helical proteins tested, BCL::Fold was able generate<8.0Å RMSD100 models for 28 cases (SSE only models) and 15 cases (complete models). Out of 16 β-proteins, this was true for all 16 cases (SSE only models) and 11 cases (complete models). For the remaining 18 αβ proteins, native-like models were generated 16 (SSE only models) and 15 cases (complete models). One of the major reasons of the difficulty experienced with a subset of these targets, as in the case of α-helical proteins 1LKIA, 1Z3XA and 2R0SA, and β-sheet proteins 1LMIA, 2QZQA and 1XAKA, can be attributed to inaccurate secondary structure predictions in terms of Q3 as well as being unable to identify one or more native SSEs.

As discussed in the introduction, BCL::Fold was designed for combination with limited experimental datasets. A version of BCL::Fold which integrates low resolution restraints from cryo-EM was previously shown to predict the correct topology for α-helical proteins [49]. Incorporation of limited experimental data from NMR and EPR experiments, folding of membrane proteins, and better reproduction of strongly bent SSEs are future directions of our research.

During review of the present manuscript, two related studies have been published. Lange et al. use the recombination of structural features that occur during the initial states of the Rosetta sampling protocol to more efficiently sample β-sheet topologies [80]. While similar in spirit, the method is restricted to<beta>sheets and structural features are gathered from models initially sampled possibly limiting the search space. This is different to our method, where improved sampling includes α-helices and β-strands and is not biased by an initial set of models. Simoncini et al. present “A Probabilistic Fragment-Based Protein Structure Prediction Algorithm” [81]. Their method “EdaFold” increases the probability for picking fragment conformations from models with low energy. This enables focused sampling of low energy conformations, assuming that they are more likely to correspond to the native protein topology. It requires that sampling trajectories communicate with each other to adjust the fragment probabilities. The amino acid sequences are still “folded” without breaks and the benchmark proteins used have only up to 128 residues, significantly less than what is used in the present study.

## Materials and Methods

### BCL::Fold Protocol and Benchmark Analysis

The flowchart of the BCL::Fold protocol is shown in Figure 2. The amino acid sequence and associated secondary structure predictions are utilized to generate a pool of SSEs (Figure 2A). The SSE pool is likely to have multiple copies for the one SSE with varying start and end points. The algorithm then selects one SSE at random from the pool and places it in the origin to start the simulation. The minimization protocol is composed of a Monte Carlo sampling algorithm (Figure 2B) coupled with knowledge-based energy potentials (Figure 2C). Once a specified number of maximum iterations are reached the minimization is ended and the model with the best energy is returned as the final model (Figure 2D). For each of the benchmark proteins, two BCL::Fold runs with 10,000 models each were completed, one using secondary structure definitions provided in the PDB files and one using the secondary structure predictions.

### Preparation of Benchmark Set

The benchmark protein set was collected using the PISCES culling server and includes 66 proteins of lengths ranging from 83 to 293 residues with<30% sequence similarity and X-ray determined structures with a resolution<2.0 Å. The set contains 66 different topologies including 31 all α-helical, 16 all β-strand, and 19 mixed αβ folds (Table 1). The primary sequence and experimental structure of the selected proteins were downloaded from the PDB [82]. The secondary structures were determined using DSSP [73], since the PDB definitions were inconsistent in some places.

### Secondary Structure Prediction and Preparation of SSE Pool

JUFO [13], [69] and PSIPRED [70] were obtained from the authors of the methods and installed locally. In addition the sequence alignment tool BLAST [83], [84] was installed locally to create the position specific scoring matrices for input to JUFO and PSIPRED. These are provided as input to the BCL::SSE application which generates a pool of likely SSEs given secondary structure prediction and BLAST profile. The prediction methods assign probabilities for each residue to be in one of three states – helix, strand or coil. BCL::SSE first generates an initial pool by taking the highest probability for each residue and assigning it the corresponding secondary structure type. A threshold of 0.5 is applied for α-helices and β-strands: if the probability is below the threshold, then the residue is assigned as a coil even if the highest probability corresponds to α-helix and β-strand. This initial pool is then refined using a Monte-Carlo based minimization composed of 1000 steps. The minimization employs moves that alter the secondary structure assignment of a single residue or divide a SSE, while the energy function used evaluates the correspondence of the secondary structure predictions to the secondary structure assignments generated (see companion BCL::Score paper for more details). For both the initial pool as well as the final pools generated by BCL::SSEs, α-helices shorter than 5 residues and β-strands shorter than 3 residues are excluded.

### SSE Pool Evaluation

Q3 is the most commonly used method for evaluating secondary structure assignments [72]. Q3 evaluates the percentage of residues with correct secondary structure assignments. However, since the actual identification of an SSE is more important than individual secondary structure assignments for BCL::Fold, we introduced additional measures: the percentage of native SSEs that were correctly identified as well as the shift, which is sum of deviations in the beginning and ends of predicted SSEs compared to native SSEs.

### Monte Carlo-based Sampling Algorithm and Temperature Control

BCL::Fold starts the minimizations with a structural model that contains a single SSE picked randomly from the pool. At each iteration, a move is selected randomly from the move set and applied to the model to produce a new structural model. The resultant model is evaluated by a consensus energy function, and whether to accept or reject this model is determined by the Metropolis criterion [85],
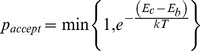
where 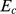 is the energy of the current model, 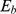 is the energy of the best model observed so far, k is a constant and T is the temperature of the system at that point. The temperature is set to 500 initially and adjusted every 10^th^ step to approach an overall cumulative move acceptance ratio for the trajectory. The target ratio for move acceptance is 0.5 in the beginning and decreases linearly to 0.2 at the end.

The evaluation of the Metropolis criterion can lead to three different results; (1) improved, if the energy of the current model is better than best the energy, (2) accepted and (3) rejected if energy of current model is worse than best energy and Metropolis criterion is used for evaluation. If this step is an “improved” state, the current model replaces the best model and minimization is continued with this model. If this step is a “rejected”, the minimization is continued with the best model observed so far. If this step is an “accepted” state, the minimization is continued on this model however the best model is not updated. An additional non-standard state “skipped” is defined if the mutate was not able to produce a new model, such as when trying to add a new SSE to a model that is already complete. In that case the energy evaluation is skipped.

### Sampling of Conformational Space

BCL::Fold explores the conformational space using a variety of moves. Each move is assigned a probability and one of them is randomly picked at each step based on these probabilities. The list of all moves utilized, their associated probabilities and descriptions can be found in Table S1 (assembly stage) and Table S2 (refinement stage). The moves can be divided into the following six categories; (1) adds, (2) removes, (3) swaps, (4) single SSE moves, (5) SSE-pair moves, (6) domain moves. For SSE, SSE-pair and domain moves, these are further categorized into specific α-helix, mixed-SSE domain, or β-sheet moves.

### Loop Building

Missing loop residues were built on to the model predicted by BCL::Fold using an in-house CCD based loop building protocol [38]. The protocol first removes a single residue from each side of all the SSEs in the model to increase the chance of being able to close the loop. Then, missing loop residues are added to the model with phi/psi angles biased by Ramachandran distribution for given amino acid type (Movies in Table S5: row “Loop grow”). The initial conformations of the residues are optimized using BCL scoring functions including amino acid clash and amino acid environment and a bias to close the chain breaks. This step ensures that initial positions can be found for all residues without causing any clashes. In the next stage, a CCD-based minimization is applied to ensure all loops are closed (Movies in Table S5: row “Loop close” and “Loop force close”).

### Composite Knowledge-based Energy Function

The composite energy function is described in detail in the companion BCL::Score manuscript. Briefly, the energy functions consists of twelve individual terms for (1) amino acid pair distance clash, (2) amino acid pair distance, (3) amino acid solvation, (4) SSE pair clash, (5) SSE pair packing, (6) β-strand pairing, (7) loop length, (8) strictly enforcing loop closure, (9) radius of gyration, (10) SSE prediction for JUFO (11) SSE prediction for PSIPRED and lastly (12) contact order. The scores for amino acid solvation and SSE predictions are also computed for the unfolded part of the protein which evaluates all residues not represented in the model, using the corresponding potentials. All scoring functions are implemented within the BCL.

All knowledge based potentials have been derived from a databank that contained 3,409 high resolution x-ray crystallography protein structures compiled using the PISCES server [86]. The collected statistical representations are converted into a free energy using the inverse Boltzmann relation and applying the appropriate normalizations. The weights for individual energy functions were optimized using a benchmark of models composed of *de novo* folded models by Rosetta [28], BCL::Fold as well as perturbed models of native structures generated by perturbation protocol within BCL. The finalized weights for energy functions used can be found in Table S3.

### Benchmark Analysis

For each BCL::Fold run of 10,000 models for each of the 66 proteins in the benchmark set, an initial filtering is done to remove any incomplete models, which was less than 3% of the models for all sets. The models produced by BCL::Fold benchmarks are evaluated by looking at the following quality measures: root-mean-square-deviation (RMSD), RMSD100 and CR. These measures are calculated over Cα atoms of all the residues in α-helices and β-strands in the models. In addition, contact order [43] values were calculated by computing the average sequence separation of contacts defined as having C_β_ (H_α2_ for Glycine) atoms within 8Å distance. Relative contact order (RCO) values were calculated by normalizing contact order values by the length of the sequence. Normalized contact orders (NCOs) were calculated by dividing the square of the contact order by the length of the sequence. An additional quality measure was developed, named contact recovery (CR), which evaluates the percentage of native contacts with a minimal sequence separation of 12 residues that are recovered in the models.

BCL::Fold models have variable SSE content, as a pool of overlapping SSEs is used. These SSEs have variable length and not each SSE is considered in each model. In result, BCL::Fold models for one and the same protein have different amino acids present. Table S4 summarizes the relative coverage of modeled residues in BCL::Fold SSE-only models. The number of modeled residues is close to the number of residues in native SSEs for all benchmark proteins. Due to over-prediction of secondary structure, SSEs that were filtered from the benchmark proteins can appear in the pool and consequently in BCL::Fold pool-SSE models. This causes the pool-coverage to be usually higher than 100%.

The variable SSE content of BCL::Fold models poses a challenge when comparing these models to the native structure and also when comparing these models to Rosetta-generated models. We considered just using residues in SSEs for both, BCL::Fold and Rosetta. However, using Rosetta secondary structure definitions puts Rosetta at a disadvantage as in particular for large proteins with β-strands secondary structure is often incompletely identified in Rosetta models due to geometric imperfections. We settled on RMSD100 and contact recovery calculations for BCL models with only the SSEs that are present in the BCL models ("incomplete") and usage of "complete" models for comparison to Rosetta.

### Protein Structure Prediction using Rosetta

The Rosetta [28] protein structure prediction program was used to predict 10,000 models for each of the benchmark proteins in order to provide a comparison for analysis of BCL::Fold. The models were produced using the *de novo* mode of Rosetta, and fragment files provided as input to Rosetta were pre-filtered to remove any fragments for homologous proteins. This was done by using the non-homolog flag of the “make fragments” Rosetta script. The resulting fragments were searched for proteins selected in more than 30% of the (overlapping) regions of the query sequence. These proteins were excluded for up to four iterations of “make fragments”. The resulting Rosetta models underwent the same analysis as the models produced by BCL::Fold. Secondary structures in Rosetta models were determined using DSSP [73] and the quality calculations were completed considering C_α_ atoms from identified α-helices and β-strands where applicable.

### BCL::Fold Availability

All components of BCL::Fold, including scoring, sampling, and clustering methods are implemented as part of the BioChemical Library (BCL) that is currently being developed in the Meiler laboratory (www.meilerlab.org). BCL::Fold is freely available for academic use along with several other components of the BCL library. Details on its usage can be found in Appendix S1.

## Supporting Information

Figure S1 **Metropolis criterion.**
(DOCX)

Figure S2 **Contact order distribution for proteins.**
(DOCX)

Figure S3 **BCL::Fold assembly runtimes for benchmark proteins.**
(DOCX)

Table S1 **Moves used in BCL::Fold assembly protocol.**
(DOCX)

Table S2 **Moves used in BCL::Fold refinement protocol.**
(DOCX)

Table S3 **Weightset for the energy function in BCL::Fold.**
(DOCX)

Table S4 **Residue coverage of BCL::Fold models relative to the number of amino acids in the native protein structure.**
(DOCX)

Table S5 **Movie file names for different proteins and minimization stages with information about the model.**
(DOCX)

Appendix S1 **BCL::Fold command line usage and file formats.**
(DOCX)

Movie S1 **1TQGA assembly of best model by RMSD100.**
(MP4)

Movie S2 **1TQGA refinement of best model by RMSD100.**
(MP4)

Movie S3 **1TQGA loop grow of best model by RMSD100.**
(MP4)

Movie S4 **1TQGA loop close of best model by RMSD100.**
(MP4)

Movie S5 **1TQGA loop force close of best model by RMSD100.**
(MP4)

Movie S6 **2RB8A assembly of best model by RMSD100.**
(MP4)

Movie S7 **2RB8A refinement of best model by RMSD100.**
(MP4)

Movie S8 **2RB8A loop grow of best model by RMSD100.**
(MP4)

Movie S9 **2RB8A loop close of best model by RMSD100.**
(MP4)

Movie S10 **2RB8A loop force close of best model by RMSD100.**
(MP4)

Movie S11 **3IV4A assembly of best model by RMSD100.**
(MP4)

Movie S12 **3IV4A refinement of best model by RMSD100.**
(MP4)

Movie S13 **3IV4A loop grow of best model by RMSD100.**
(MP4)

Movie S14 **3IV4A loop close of best model by RMSD100.**
(MP4)

Movie S15 **3IV4A loop force close of best model by RMSD100.**
(MP4)

## References

[1] Westbrook J, Feng Z, Chen L, Yang H, Berman HM (2003) The Protein Data Bank and structural genomics. Nucleic Acids Res 31: 489–491. 12520059 10.1093/nar/gkg068 PMC165515

[2] Berman HM, Battistuz T, Bhat TN, Bluhm WF, Bourne PE, et al (2002) The Protein Data Bank. Acta Crystallogr D Biol Crystallogr 58: 899–907. 12037327 10.1107/s0907444902003451

[3] Daga PR, Patel RY, Doerksen RJ (2010) Template-based protein modeling: recent methodological advances. Current topics in medicinal chemistry 10: 84–94. 19929829 10.2174/156802610790232314 PMC5943704

[4] Stevens RC, Yokoyama S, Wilson IA (2001) Global efforts in structural genomics. Science 294: 89–92. 11588249 10.1126/science.1066011

[5] Lesley SA, Kuhn P, Godzik A, Deacon AM, Mathews I, et al (2002) Structural genomics of the Thermotoga maritima proteome implemented in a high-throughput structure determination pipeline. Proc Natl Acad Sci U S A 99: 11664–11669. 12193646 10.1073/pnas.142413399 PMC129326

[6] DiMaio F, Terwilliger TC, Read RJ, Wlodawer A, Oberdorfer G, et al (2011) Improved molecular replacement by density- and energy-guided protein structure optimization. Nature 473: 540–543. 21532589 10.1038/nature09964 PMC3365536

[7] Bill RM, Henderson PJ, Iwata S, Kunji ER, Michel H, et al (2011) Overcoming barriers to membrane protein structure determination. Nature biotechnology 29: 335–340. 10.1038/nbt.1833 21478852

[8] Oberai A, Ihm Y, Kim S, Bowie JU (2006) A limited universe of membrane protein families and folds. Protein Sci 15: 1723–1734. 16815920 10.1110/ps.062109706 PMC2242558

[9] Alber F, Dokudovskaya S, Veenhoff LM, Zhang W, Kipper J, et al (2007) Determining the architectures of macromolecular assemblies. Nature 450: 683–694. 18046405 10.1038/nature06404

[10] Yooseph S, Sutton G, Rusch DB, Halpern AL, Williamson SJ, et al (2007) The Sorcerer II Global Ocean Sampling expedition: expanding the universe of protein families. PLoS biology 5: e16. 17355171 10.1371/journal.pbio.0050016 PMC1821046

[11] Rost B (1996) PHD: predicting one-dimensional protein structure by profile-based neural networks. Methods Enzymol 266: 525–539. 8743704 10.1016/s0076-6879(96)66033-9

[12] Karplus K, Sjolander K, Barrett C, Cline M, Haussler D, et al.. (1997) Predicting protein structure using hidden Markov models. Proteins Suppl 1: 134–139. 10.1002/(sici)1097-0134(1997)1+<134::aid-prot18>3.3.co;2-q 9485505

[13] Meiler J, Baker D (2003) Coupled prediction of protein secondary and tertiary structure. Proceedings of the National Academy of Sciences of the United States of America 100: 12105–12110. 14528006 10.1073/pnas.1831973100 PMC218720

[14] Ward JJ, McGuffin LJ, Buxton BF, Jones DT (2003) Secondary structure prediction with support vector machines. Bioinformatics 19: 1650–1655. 12967961 10.1093/bioinformatics/btg223

[15] Kuhn M, Meiler J, Baker D (2004) Strand-loop-strand motifs: prediction of hairpins and diverging turns in proteins. Proteins 54: 282–288. 14696190 10.1002/prot.10589

[16] Jones DT, Ward JJ (2003) Prediction of disordered regions in proteins from position specific score matrices. Proteins 53 Suppl 6573–578. 14579348 10.1002/prot.10528

[17] Linding R, Jensen LJ, Diella F, Bork P, Gibson TJ, et al (2003) Protein Disorder Prediction: Implications for Structural Proteomics. Structure 11: 1453–1459. 14604535 10.1016/j.str.2003.10.002

[18] Grana O, Baker D, MacCallum RM, Meiler J, Punta M, et al (2005) CASP6 assessment of contact prediction. Proteins 61 Suppl 7214–224. 16187364 10.1002/prot.20739

[19] Liu J, Rost B (2001) Comparing function and structure between entire proteomes. Protein Sci 10: 1970–1979. 11567088 10.1110/ps.10101 PMC2374214

[20] Galzitskaya OV, Melnik BS (2003) Prediction of protein domain boundaries from sequence alone. Protein Sci 12: 696–701. 12649427 10.1110/ps.0233103 PMC2323849

[21] Chivian D, Kim DE, Malmstrom L, Schonbrun J, Rohl CA, et al (2005) Prediction of CASP6 structures using automated Robetta protocols. Proteins 61 Suppl 7157–166. 16187358 10.1002/prot.20733

[22] Valencia A, Pazos F (2002) Computational methods for the prediction of protein interactions. Curr Opin Struct Biol 12: 368–373. 12127457 10.1016/s0959-440x(02)00333-0

[23] Ben-Hur A, Noble WS (2005) Kernel methods for predicting protein-protein interactions. Bioinformatics 21 Suppl 1i38–i46. 15961482 10.1093/bioinformatics/bti1016

[24] Rost B (2003) Prediction in 1D: secondary structure, membrane helices, and accessibility. Methods Biochem Anal 44: 559–587. 12647405

[25] Rost B (2001) Review: protein secondary structure prediction continues to rise. J Struct Biol 134: 204–218. 11551180 10.1006/jsbi.2001.4336

[26] Bradley P, Malmstrom L, Qian B, Schonbrun J, Chivian D, et al (2005) Free modeling with Rosetta in CASP6. Proteins 61 Suppl 7128–134. 16187354 10.1002/prot.20729

[27] Bradley P, Chivian D, Meiler J, Misura K, Wedemeyer W, et al (2003) Rosetta in CASP5: Progress in ab initio protein structure prediction. Proteins: Struct, Funct, Genet 53: 457–468.14579334

[28] Simons KT, Kooperberg C, Huang E, Baker D (1997) Assembly of Protein Tertiary Structures from Fragments with Similar Local Sequences using Simulated Annealing and Bayesian Scoring Functions. J Mol Biol 268: 209–225. 9149153 10.1006/jmbi.1997.0959

[29] Bonneau R, Strauss CEM, Rohl C, Chivian D, Bradley P, et al (2002) De Novo Prediction of Three-dimensional Structures for Major Protein Families. J Mol Biol 322: 65–78. 12215415 10.1016/s0022-2836(02)00698-8

[30] Zhou H, Pandit SB, Skolnick J (2009) Performance of the Pro-sp3-TASSER server in CASP8. Proteins 77 Suppl 9123–127. 19639638 10.1002/prot.22501 PMC2785221

[31] Zhou H, Skolnick J (2007) Ab initio protein structure prediction using chunk-TASSER. Biophys J 93: 1510–1518. 17496016 10.1529/biophysj.107.109959 PMC1948038

[32] Dahiyat BI, Mayo SL (1997) De novo protein design: fully automated sequence selection. Science 278: 82–87. 9311930 10.1126/science.278.5335.82

[33] Kuhlman B, Baker D (2000) Native protein sequences are close to optimal for their structures. Proc Natl Acad Sci U S A 97: 10383–10388. 10984534 10.1073/pnas.97.19.10383 PMC27033

[34] Dunbrack RL Jr (2002) Rotamer libraries in the 21st century. Curr Opin Struct Biol 12: 431–440. 12163064 10.1016/s0959-440x(02)00344-5

[35] Bradley P, Misura KM, Baker D (2005) Toward high-resolution de novo structure prediction for small proteins. Science 309: 1868–1871. 16166519 10.1126/science.1113801

[36] Smith JA, Vanoye CG, George AL Jr, Meiler J, Sanders CR (2007) Structural models for the KCNQ1 voltage-gated potassium channel. Biochemistry 46: 14141–14152. 17999538 10.1021/bi701597s PMC2565492

[37] Eswar N, Webb B, Marti-Renom MA, Madhusudhan MS, Eramian D, et al.. (2006) Comparative protein structure modeling using Modeller. Current protocols in bioinformatics/editoral board, Andreas D Baxevanis [et al] Chapter 5: Unit 5 6. 10.1002/0471250953.bi0506s15 PMC4186674 18428767

[38] Canutescu AA, Dunbrack RL (2003) Cyclic coordinate descent: A robotics algorithm for protein loop closure. Protein Sci 12: 963–972. 12717019 10.1110/ps.0242703 PMC2323867

[39] Sali A, Blundell TL (1993) Comparitive Protein Modelling by Satisfaction of Spatial Restraints. J Mol Biol 234: 779–815. 8254673 10.1006/jmbi.1993.1626

[40] Rohl CA, Strauss CE, Chivian D, Baker D (2004) Modeling structurally variable regions in homologous proteins with rosetta. Proteins 55: 656–677. 15103629 10.1002/prot.10629

[41] Baker D (2000) A surprising simplicity to protein folding. Nature 405: 39–42. 10811210 10.1038/35011000

[42] Grantcharova V, Alm EJ, Baker D, Horwich AL (2001) Mechanisms of protein folding. Curr Opin Struct Biol 11: 70–82. 11179895 10.1016/s0959-440x(00)00176-7

[43] Bonneau R, Ruczinski I, Tsai J, Baker D (2002) Contact order and ab initio protein structure prediction. Protein Sci 11: 1937–1944. 12142448 10.1110/ps.3790102 PMC2373674

[44] Plaxco KW, Simons KT, Ruczinski I, Baker D (2000) Topology, stability, sequence, and length: defining the determinants of two-state protein folding kinetics. Biochemistry 39: 11177–11183. 10985762 10.1021/bi000200n

[45] Lindert S, Silvestry M, Mullen TM, Nemerow GR, Stewart PL (2009) Cryo-electron microscopy structure of an adenovirus-integrin complex indicates conformational changes in both penton base and integrin. Journal of virology 83: 11491–11501. 19726496 10.1128/JVI.01214-09 PMC2772687

[46] Zimmer J, Nam Y, Rapoport TA (2008) Structure of a complex of the ATPase SecA and the protein-translocation channel. Nature 455: 936–943. 18923516 10.1038/nature07335 PMC7164768

[47] Sibanda BL, Chirgadze DY, Blundell TL (2010) Crystal structure of DNA-PKcs reveals a large open-ring cradle comprised of HEAT repeats. Nature 463: 118–121. 20023628 10.1038/nature08648 PMC2811870

[48] Fleishman SJ, Harrington SE, Enosh A, Halperin D, Tate CG, et al (2006) Quasi-symmetry in the cryo-EM structure of EmrE provides the key to modeling its transmembrane domain. Journal of molecular biology 364: 54–67. 17005200 10.1016/j.jmb.2006.08.072

[49] Lindert S, Staritzbichler R, Wotzel N, Karakas M, Stewart PL, et al (2009) EM-fold: De novo folding of alpha-helical proteins guided by intermediate-resolution electron microscopy density maps. Structure 17: 990–1003. 19604479 10.1016/j.str.2009.06.001 PMC3760413

[50] Skrisovska L, Schubert M, Allain FH (2010) Recent advances in segmental isotope labeling of proteins: NMR applications to large proteins and glycoproteins. Journal of biomolecular NMR 46: 51–65. 19690964 10.1007/s10858-009-9362-7

[51] Ganguly S, Weiner BE, Meiler J (2011) Membrane protein structure determination using paramagnetic tags. Structure 19: 441–443. 21481766 10.1016/j.str.2011.03.008 PMC3754786

[52] Chen H, Ji F, Olman V, Mobley CK, Liu Y, et al (2011) Optimal mutation sites for PRE data collection and membrane protein structure prediction. Structure 19: 484–495. 21481772 10.1016/j.str.2011.02.002 PMC3099474

[53] Wang X, Watson C, Sharp JS, Handel TM, Prestegard JH (2011) Oligomeric structure of the chemokine CCL5/RANTES from NMR, MS, and SAXS data. Structure 19: 1138–1148. 21827949 10.1016/j.str.2011.06.001 PMC3159919

[54] McHaourab HS, Steed PR, Kazmier K (2011) Toward the Fourth Dimension of Membrane Protein Structure: Insight into Dynamics from Spin-Labeling EPR Spectroscopy. Structure 19: 1549–1561. 22078555 10.1016/j.str.2011.10.009 PMC3224804

[55] Van Horn WD, Kim HJ, Ellis CD, Hadziselimovic A, Sulistijo ES, et al (2009) Solution nuclear magnetic resonance structure of membrane-integral diacylglycerol kinase. Science 324: 1726–1729. 19556511 10.1126/science.1171716 PMC2764269

[56] Singh P, Panchaud A, Goodlett DR (2010) Chemical cross-linking and mass spectrometry as a low-resolution protein structure determination technique. Analytical chemistry 82: 2636–2642. 20210330 10.1021/ac1000724

[57] Kalkhof S, Haehn S, Paulsson M, Smyth N, Meiler J, et al (2010) Computational modeling of laminin N-terminal domains using sparse distance constraints from disulfide bonds and chemical cross-linking. Proteins 78: 3409–3427. 20939100 10.1002/prot.22848 PMC5079110

[58] Young MM, Tang N, Hempel JC, Oshiro CM, Taylor EW, et al (2000) High throughput protein fold identification by using experimental constraints derived from intramolecular cross-links and mass spectrometry. Proceedings of the National Academy of Sciences of the United States of America 97: 5802–5806. 10811876 10.1073/pnas.090099097 PMC18514

[59] Qian B, Raman S, Das R, Bradley P, McCoy AJ, et al (2007) High-resolution structure prediction and the crystallographic phase problem. Nature 450: 259–264. 17934447 10.1038/nature06249 PMC2504711

[60] Meiler J, Baker D (2003) Rapid Protein Structure Elucidation Utilizing Unassigned NMR Data. PNAS 100: 15404–15409. 14668443 10.1073/pnas.2434121100 PMC307580

[61] Raman S, Lange OF, Rossi P, Tyka M, Wang X, et al (2010) NMR structure determination for larger proteins using backbone-only data. Science 327: 1014–1018. 20133520 10.1126/science.1183649 PMC2909653

[62] Alexander N, Bortolus M, Al-Mestarihi A, McHaourab H, Meiler J (2008) De novo high-resolution protein structure determination from sparse spin-labeling EPR data. Structure 16: 181–195. 18275810 10.1016/j.str.2007.11.015 PMC2390841

[63] Kazmier K, Alexander NS, Meiler J, McHaourab HS (2011) Algorithm for selection of optimized EPR distance restraints for de novo protein structure determination. Journal of structural biology 173: 549–557. 21074624 10.1016/j.jsb.2010.11.003 PMC3073550

[64] Hirst SJ, Alexander N, McHaourab HS, Meiler J (2011) RosettaEPR: an integrated tool for protein structure determination from sparse EPR data. Journal of structural biology 173: 506–514. 21029778 10.1016/j.jsb.2010.10.013 PMC3040274

[65] Hussain SA, Carafoli F, Hohenester E (2011) Determinants of laminin polymerization revealed by the structure of the alpha5 chain amino-terminal region. EMBO reports 12: 276–282. 21311558 10.1038/embor.2011.3 PMC3059903

[66] Kolinski A, Skolnick J (1998) Assembly of protein structure from sparse experimental data: an efficient Monte Carlo model. Proteins 32: 475–494. 9726417

[67] Latek D, Kolinski A (2011) CABS-NMR–De novo tool for rapid global fold determination from chemical shifts, residual dipolar couplings and sparse methyl-methyl NOEs. Journal of computational chemistry 32: 536–544. 20806263 10.1002/jcc.21640

[68] Barth P, Wallner B, Baker D (2009) Prediction of membrane protein structures with complex topologies using limited constraints. Proceedings of the National Academy of Sciences of the United States of America 106: 1409–1414. 19190187 10.1073/pnas.0808323106 PMC2635801

[69] Meiler J, Muller M, Zeidler A, Schmaschke F (2001) Generation and evaluation of dimension-reduced amino acid parameter representations by artificial neural networks. Journal of Molecular Modeling 7: 360–369.

[70] Jones DT (1999) Protein secondary structure prediction based on position-specific scoring matrices. Journal of Molecular Biology 292: 195–202. 10493868 10.1006/jmbi.1999.3091

[71] Chandonia JM, Karplus M (1999) New methods for accurate prediction of protein secondary structure. Proteins 35: 293–306. 10328264

[72] Rost B, Sander C, Schneider R (1994) Redefining the Goals of Protein Secondary Structure Prediction. J Mol Biol 235: 13–26. 8289237 10.1016/s0022-2836(05)80007-5

[73] Kabsch W, Sander C (1983) Dictionary of Protein Secondary Structure: Pattern Recognition of Hydrogen-Bonded and Geometrical Features. Biopolymers 22: 2577–2637. 6667333 10.1002/bip.360221211

[74] Carugo O, Pongor S (2001) A normalized root-mean-square distance for comparing protein three-dimensional structures. Protein science : a publication of the Protein Society 10: 1470–1473. 11420449 10.1110/ps.690101 PMC2374114

[75] Moult J (2005) A decade of CASP: progress, bottlenecks and prognosis in protein structure prediction. Current opinion in structural biology 15: 285–289. 15939584 10.1016/j.sbi.2005.05.011

[76] Moult J, Hubbard T, Bryant SH, Fidelis K, Pedersen JT (1997) Critical assessment of methods of protein structure prediction (CASP): round II. Proteins Suppl 1: 2–6. 9485489

[77] Kryshtafovych A, Krysko O, Daniluk P, Dmytriv Z, Fidelis K (2009) Protein structure prediction center in CASP8. Proteins 77 Suppl 95–9. 19722263 10.1002/prot.22517 PMC2863353

[78] Fiser A, Sali A (2003) Modeller: generation and refinement of homology-based protein structure models. Methods in enzymology 374: 461–491. 14696385 10.1016/S0076-6879(03)74020-8

[79] Kinch LN, Shi S, Cheng H, Cong Q, Pei J, et al (2011) CASP9 target classification. Proteins 79 Suppl 1021–36. 21997778 10.1002/prot.23190 PMC3226894

[80] Lange OF, Baker D (2012) Resolution-adapted recombination of structural features significantly improves sampling in restraint-guided structure calculation. Proteins 80: 884–895. 22423358 10.1002/prot.23245 PMC3310173

[81] Simoncini D, Berenger F, Shrestha R, Zhang KY (2012) A probabilistic fragment-based protein structure prediction algorithm. PloS one 7: e38799. 22829868 10.1371/journal.pone.0038799 PMC3400640

[82] Bernstein FC, Koetzle TF, Williams GJ, Meyer EF Jr, Brice MD, et al (1977) The Protein Data Bank: a computer-based archival file for macromolecular structures. J Mol Biol 112: 535–542. 875032 10.1016/s0022-2836(77)80200-3

[83] Altschul SF, Gish W, Miller W, Myers EW, Lipman DJ (1990) Basic local alignment search tool. J Mol Biol 215: 403–410. 2231712 10.1016/S0022-2836(05)80360-2

[84] Altschul SF, Madden TL, Schäffer AA, Zhang J, Zhang Z, et al (1997) Gapped BLAST and PSI-BLAST: a new generation of protein database search programs. Nucleic Acids Res 25: 3389–3402. 9254694 10.1093/nar/25.17.3389 PMC146917

[85] Metropolis NaU (1949) S (1949) The Monte Carlo Method. J Amer Stat Assoc 44: 335–341. 18139350 10.1080/01621459.1949.10483310

[86] Wang GL, Dunbrack RL (2005) PISCES: recent improvements to a PDB sequence culling server. Nucleic Acids Research 33: W94–W98. 15980589 10.1093/nar/gki402 PMC1160163

