# Supplementary material for: BCL::Fold - De Novo Prediction of Complex and Large Protein Topologies by Assembly of Secondary Structure Elements
Source: PLoS One. 2012 Nov 16;7(11):e49240. doi: 10.1371/journal.pone.0049240 (PMC3500284; doi:10.1371/journal.pone.0049240)
Supplement: Figure S1 — Metropolis criterion. (DOCX) [file pone.0049240.s001.docx]

BCL::Fold uses a Monte Carlo Metropolis energy minimization. Figure S1 illustrates how the Metropolis criterion is implemented in BCL::Fold.

**
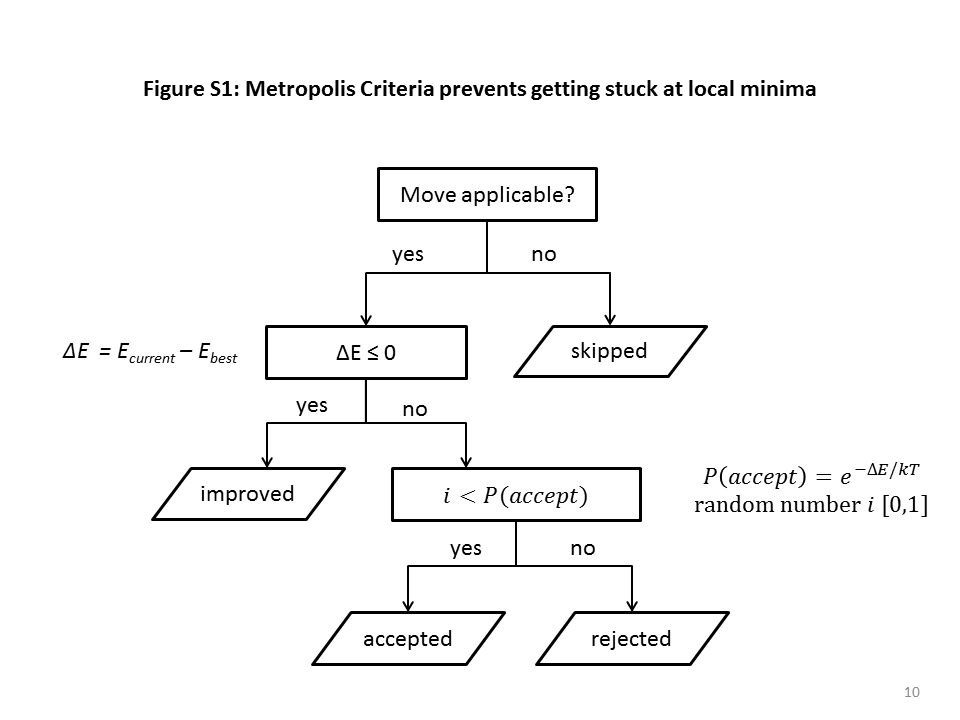
**

**Figure S1: Metropolis criterion**

At each step, the result is evaluated and the step is assigned one of four possible outcomes; skipped, improved, accepted, and rejected. The first check determines whether the move was applicable or not. In cases where the SSEs required for the move are not yet added to the model, the step is determined to be as skipped. If the move was successfully applied, then the energy difference to the last improved model is calculated, if the energy has improved (became lower), then the step is assigned as improved. If the energy increased, then a random number is used to determine whether the move should still be accepted. The acceptance ratio for this purpose is biased by the amount of the increase in the energy.

Figure S2 rationalizes the introduction of the normalized contact order measure (NCO) measure as relative contact order (RCO) is dependent on chain length. The heat map shows the distribution of contact order with respect to sequence lengths for ~4000 culled native proteins.
