# Supplementary material for: BCL::Fold - De Novo Prediction of Complex and Large Protein Topologies by Assembly of Secondary Structure Elements
Source: PLoS One. 2012 Nov 16;7(11):e49240. doi: 10.1371/journal.pone.0049240 (PMC3500284; doi:10.1371/journal.pone.0049240)
Supplement: Figure S2 — Contact order distribution for proteins. (DOCX) [file pone.0049240.s002.docx]

Figure S2 rationalizes the introduction of the normalized contact order measure (NCO) measure as relative contact order (RCO) is dependent on chain length. The heat map shows the distribution of contact order with respect to sequence lengths for ~4000 culled native proteins.

**
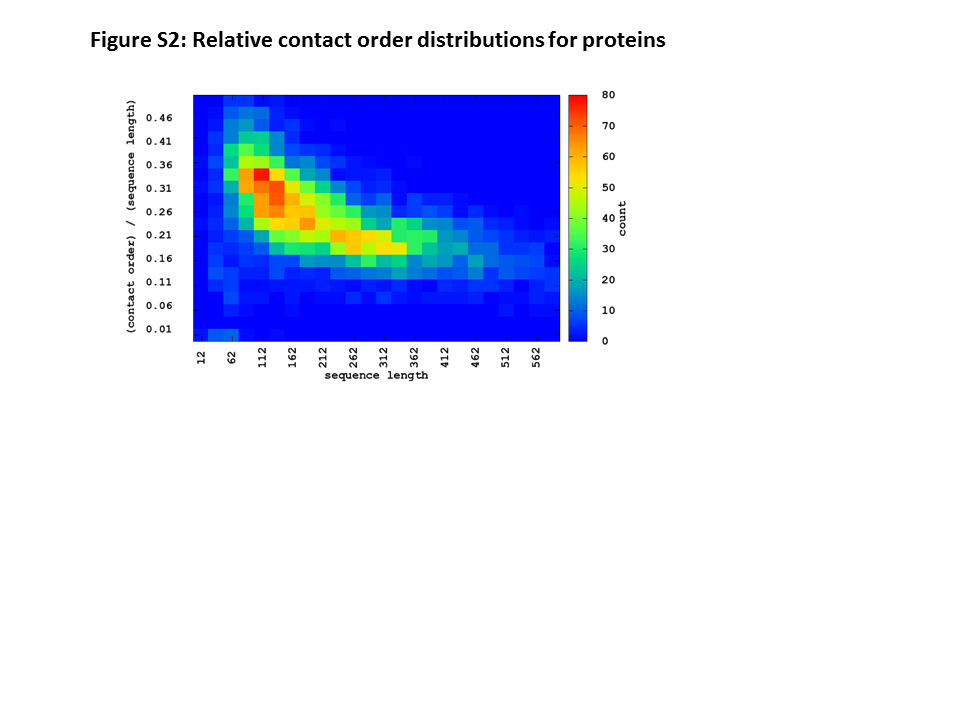
**

**Figure S2: Contact order distribution for proteins**

Figure S3 plots the average runtimes in minutes per model (left axis) as a bar graph for each of the 66 benchmark proteins. The number of amino acids for each benchmark protein is indicated by the line (right axis). Simulation time increases linearly with sequence length.
