# Supplementary material for: BCL::Fold - De Novo Prediction of Complex and Large Protein Topologies by Assembly of Secondary Structure Elements
Source: PLoS One. 2012 Nov 16;7(11):e49240. doi: 10.1371/journal.pone.0049240 (PMC3500284; doi:10.1371/journal.pone.0049240)
Supplement: Figure S3 — BCL::Fold assembly runtimes for benchmark proteins. (DOCX) [file pone.0049240.s003.docx]

**
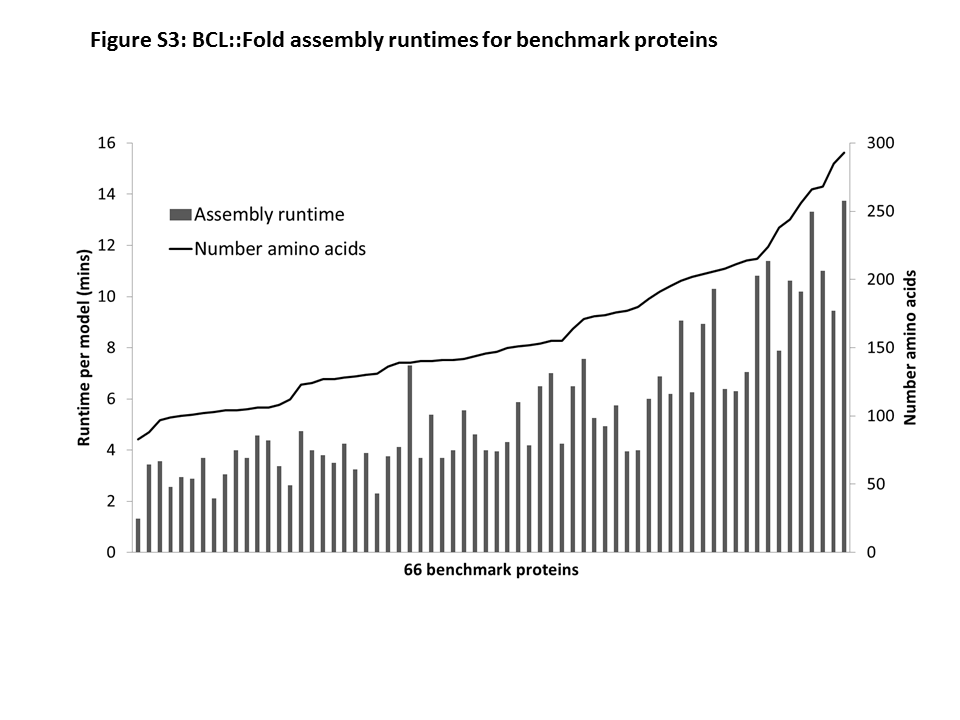
**

**Figure S3: BCL::Fold assembly runtimes for benchmark proteins**

Table S1 lists all moves used in BCL::Fold assembly protocol along with the subcategory they belong to. This is followed by counts and percentages on minimization steps where each move was used along with what kind of Metropolis result these steps have led to; total number of steps used in(N_T_), number and percentage of improved steps(N_I_ and P_I_), accepted steps (N_A_ and P_A_), rejected steps (N_R_ and P_R_), skipped steps(N_S_ and P_S_). This is followed by Δ_MEAN,_ which represents the average energy decrease in the energy from the last improved model for cases where the move has led to an improved step. The last column gives a short description of what each move does.
