# Supplementary material for: BCL::Fold - De Novo Prediction of Complex and Large Protein Topologies by Assembly of Secondary Structure Elements
Source: PLoS One. 2012 Nov 16;7(11):e49240. doi: 10.1371/journal.pone.0049240 (PMC3500284; doi:10.1371/journal.pone.0049240)
Supplement: Table S1 — Moves used in BCL::Fold assembly protocol. (DOCX) [file pone.0049240.s004.docx]

Table S1 lists all moves used in BCL::Fold assembly protocol along with the subcategory they belong to. This is followed by counts and percentages on minimization steps where each move was used along with what kind of Metropolis result these steps have led to; total number of steps used in(N_T_), number and percentage of improved steps(N_I_ and P_I_), accepted steps (N_A_ and P_A_), rejected steps (N_R_ and P_R_), skipped steps(N_S_ and P_S_). This is followed by Δ_MEAN,_ which represents the average energy decrease in the energy from the last improved model for cases where the move has led to an improved step. The last column gives a short description of what each move does.

| **Move#** | **Move** | **Type** | **N_total_** | **N_improved_** | **N_accepted_** | **N_rejected_** | **N_skipped_** | **%_improved_** | **%_accepted_** | **%_rejected_** | **%_skipped_** | **Δ_mean_** | **description** |
| --- | --- | --- | --- | --- | --- | --- | --- | --- | --- | --- | --- | --- | --- |
| 1 | add_sse_next_to_sse | add | 136680 | 2732 | 9259 | 21867 | 102822 | 2.0 | 6.8 | 16.0 | 75.2 | -418.2 | add an SSE from the pool to the model using preferred orientations |
| 2 | add_sse_short_loop | add | 45313 | 1338 | 3236 | 6391 | 34348 | 3.0 | 7.1 | 14.1 | 75.8 | -362.5 | add an SSE from the pool next to an SSE which is a neighbor in sequence |
| 3 | add_strand_next_to_sheet | add | 60945 | 1276 | 1827 | 2957 | 54885 | 2.1 | 3.0 | 4.9 | 90.1 | -355.8 | add a strand to sheet as the edge strand |
| 4 | remove_random | remove | 40322 | 66 | 9180 | 30933 | 143 | 0.2 | 22.8 | 76.7 | 0.4 | -171.2 | remove a randomly determined SSE from the model |
| 5 | remove_unpaired_strand | remove | 40341 | 42 | 2998 | 5503 | 31798 | 0.1 | 7.4 | 13.6 | 78.8 | -232.5 | locate and remove an unpaired strand from the model |
| 6 | swap_sse_with_pool | swap | 54057 | 572 | 2744 | 5464 | 45277 | 1.1 | 5.1 | 10.1 | 83.8 | -254.7 | swap an SSE in the model with an SSE from the pool |
| 7 | swap_sse_with_pool_overlap | swap | 54238 | 2270 | 18203 | 32849 | 916 | 4.2 | 33.6 | 60.6 | 1.7 | -89.9 | swap an SSE in the model with an SSE from the pool which overlaps |
| 8 | swap_sses | swap | 215542 | 1639 | 49007 | 160826 | 4070 | 0.8 | 22.7 | 74.6 | 1.9 | -107.9 | swap locations of two SSEs in the model |
| 9 | sse_furthest_move_next | SSE | 28436 | 332 | 5532 | 22572 | 0 | 1.2 | 19.5 | 79.4 | 0.0 | -160.9 | Locate the SSE in the model furthest from the center and re-place it next to another SSE |
| 10 | sse_move_next | SSE | 28489 | 150 | 4948 | 23391 | 0 | 0.5 | 17.4 | 82.1 | 0.0 | -127.9 | Locate a random SSE in the model and re-place it next to another SSE |
| 11 | sse_move_short_loop | SSE | 28725 | 236 | 5125 | 19980 | 3384 | 0.8 | 17.8 | 69.6 | 11.8 | -153.2 | Locate a random SSE in the model and re-place it next to an SSE which has a short loop to it |
| 12 | sse_resize_cterm | SSE | 14169 | 1101 | 4435 | 6877 | 1756 | 7.8 | 31.3 | 48.5 | 12.4 | -56.4 | Extend/shrink a random SSE by 1 to 3 residues from C terminal end |
| 13 | sse_resize_nterm | SSE | 14332 | 1075 | 4552 | 6960 | 1745 | 7.5 | 31.8 | 48.6 | 12.2 | -56.7 | Extend/shrink a random SSE by 1 to 3 residues from N terminal end |
| 14 | sse_rotate_large | SSE | 28245 | 391 | 6575 | 21279 | 0 | 1.4 | 23.3 | 75.3 | 0.0 | -51.0 | Rotate an SSE by 15 to 45 degrees in any direction |
| 15 | sse_rotate_x_large | SSE | 9365 | 260 | 2364 | 6741 | 0 | 2.8 | 25.2 | 72.0 | 0.0 | -38.0 | Rotate an SSE by 0 to 45 degrees around X axis |
| 16 | sse_rotate_y_large | SSE | 9529 | 456 | 2839 | 6234 | 0 | 4.8 | 29.8 | 65.4 | 0.0 | -76.0 | Rotate an SSE by up to 45 degrees around Y axis |
| 17 | sse_rotate_z_large | SSE | 9502 | 904 | 3785 | 4813 | 0 | 9.5 | 39.8 | 50.7 | 0.0 | -19.2 | Rotate an SSE by up to 45 degrees around Z axis |
| 18 | sse_split_JUFO | SSE | 19644 | 215 | 4396 | 12110 | 2923 | 1.1 | 22.4 | 61.7 | 14.9 | -69.1 | Split a long SSE ( >14 residues for helices, > 8 residues for strands) into two shorter SSE by removing the residue in the SSE with the lowest JUFO prediction for the associated SS type |
| 19 | sse_split_PSIPRED | SSE | 19624 | 256 | 4372 | 11893 | 3103 | 1.3 | 22.3 | 60.6 | 15.8 | -59.5 | Same as sse_split_JUFO, but uses PSIPRED predictions instead |
| 20 | sse_translate_large | SSE | 28450 | 195 | 6104 | 22151 | 0 | 0.7 | 21.5 | 77.9 | 0.0 | -77.6 | Translate an SSE 2 to 6Å along any direction |
| 21 | sse_translate_x_large | SSE | 9396 | 202 | 2667 | 6527 | 0 | 2.2 | 28.4 | 69.5 | 0.0 | -64.2 | Translate an SSE up to 6Å along X axis |
| 22 | sse_translate_y_large | SSE | 9493 | 190 | 2150 | 7153 | 0 | 2.0 | 22.7 | 75.4 | 0.0 | -45.5 | Translate an SSE up to 6Å along Y axis |
| 23 | sse_translate_z_large | SSE | 9446 | 694 | 3655 | 5097 | 0 | 7.4 | 38.7 | 54.0 | 0.0 | -34.6 | Translate an SSE up to 6Å along Z axis |
| 24 | sse_transform_large | SSE | 56882 | 248 | 10963 | 45671 | 0 | 0.4 | 19.3 | 80.3 | 0.0 | -76.2 | Transform an SSE in any direction by up to 2Å translation and 15 degree rotation |
| 25 | helix_flip_xy | α-helix | 33492 | 1028 | 10259 | 21968 | 237 | 3.1 | 30.6 | 65.6 | 0.7 | -69.7 | Rotate a randomly picked helix by 180 degrees around X or Y axis |
| 26 | helix_flip_z | α-helix | 33826 | 1473 | 12322 | 19780 | 251 | 4.4 | 36.4 | 58.5 | 0.7 | -54.4 | Rotate a randomly picked helix by 180 degrees around Z axis |
| 27 | helix_furthest_move_next | α-helix | 33590 | 405 | 6958 | 26026 | 201 | 1.2 | 20.7 | 77.5 | 0.6 | -163.0 | Locate the helix in the model furthest from the center and re-place it next to another SSE |
| 28 | helix_move_next | α-helix | 33229 | 202 | 6183 | 26624 | 220 | 0.6 | 18.6 | 80.1 | 0.7 | -142.5 | Locate a random SSE in the model and re-place it next to another SSE |
| 29 | helix_move_short_loop | α-helix | 33256 | 321 | 6239 | 22364 | 4332 | 1.0 | 18.8 | 67.3 | 13.0 | -193.5 | Locate a random SSE in the model and re-place it next to an SSE which has a short loop to it |
| 30 | helix_translate_xy_large | α-helix | 33399 | 565 | 9300 | 23324 | 210 | 1.7 | 27.9 | 69.8 | 0.6 | -64.6 | Translate an helix 2 to 4Å along x axis and y axis |
| 31 | helix_translate_z_large | α-helix | 33459 | 3070 | 12898 | 17270 | 221 | 9.2 | 38.6 | 51.6 | 0.7 | -30.3 | Translate an helix up to 4Å along z axis |
| 32 | helix_rotate_xy_large | α-helix | 33346 | 704 | 9219 | 23222 | 201 | 2.1 | 27.7 | 69.6 | 0.6 | -45.4 | Rotate an helix 15 to 45 degrees around x axis and y axis |
| 33 | helix_rotate_z_large | α-helix | 33539 | 4956 | 14400 | 13915 | 268 | 14.8 | 42.9 | 41.5 | 0.8 | -19.8 | Rotate an helix 15 to 45 degrees around z axis |
| 34 | helix_transform_xy_large | α-helix | 33435 | 320 | 7988 | 24909 | 218 | 1.0 | 23.9 | 74.5 | 0.7 | -68.6 | Transform a helix by 2 to 4A translation and 15 to 45 degrees rotation in x axis and y axis |
| 35 | helix_transform_z_large | α-helix | 33453 | 1638 | 11573 | 20025 | 217 | 4.9 | 34.6 | 59.9 | 0.7 | -47.1 | Transform a helix by 2 to 4A translation and 15 to 45 degrees rotation in z axis |
| 36 | strand_flip_x | β-strand | 34875 | 633 | 9966 | 23602 | 674 | 1.8 | 28.6 | 67.7 | 1.9 | -90.6 | Rotate a randomly picked strand by 180 degrees around X axis |
| 37 | strand_flip_y | β-strand | 35082 | 549 | 9612 | 24232 | 689 | 1.6 | 27.4 | 69.1 | 2.0 | -90.9 | Rotate a randomly picked strand by 180 degrees around Y axis |
| 38 | strand_flip_z | β-strand | 34732 | 3163 | 16662 | 14216 | 691 | 9.1 | 48.0 | 40.9 | 2.0 | -18.6 | Rotate a randomly picked strand by 180 degrees around Z axis |
| 39 | strand_furthest_move_next | β-strand | 34969 | 278 | 6012 | 28000 | 679 | 0.8 | 17.2 | 80.1 | 1.9 | -136.0 | Locate the strand in the model furthest from the center and re-place it next to another SSE |
| 40 | strand_furthest_move_sheet | β-strand | 34723 | 466 | 7266 | 22450 | 4541 | 1.3 | 20.9 | 64.7 | 13.1 | -137.5 | Locate the strand in the model furthest from the center and re-place it next to a sheet |
| 41 | strand_move_next | β-strand | 34589 | 175 | 5223 | 28509 | 682 | 0.5 | 15.1 | 82.4 | 2.0 | -147.0 | Locate a random strand in the model and re-place it next to another SSE |
| 42 | strand_move_sheet | β-strand | 35111 | 274 | 6182 | 23958 | 4697 | 0.8 | 17.6 | 68.2 | 13.4 | -140.1 | Locate a random strand in the model and re-place it next to a sheet |
| 43 | strand_translate_z_large | β-strand | 34897 | 3221 | 14769 | 16275 | 632 | 9.2 | 42.3 | 46.6 | 1.8 | -24.7 | Translate a strand 2 to 4Å along z axis |
| 44 | ssepair_translate_large | SSE pair | 70497 | 826 | 8559 | 19315 | 41797 | 1.2 | 12.1 | 27.4 | 59.3 | -49.5 | Locate two packed SSEs, translate one of them 1 to 3Å along the packing axis |
| 45 | ssepair_translate_no_hinge_large | SSE pair | 70240 | 188 | 6514 | 21985 | 41553 | 0.3 | 9.3 | 31.3 | 59.2 | -55.1 | Locate two packed SSEs, translate one of them 2 to 4Å in any axis of the other one |
| 46 | ssepair_rotate_large | SSE pair | 70359 | 1010 | 7737 | 19912 | 41700 | 1.4 | 11.0 | 28.3 | 59.3 | -48.8 | Locate two packed SSEs, rotate one of them 10 to 45 degrees around the packing axis |
| 47 | ssepair_transform_large | SSE pair | 70300 | 342 | 6660 | 21607 | 41691 | 0.5 | 9.5 | 30.7 | 59.3 | -61.5 | Locate two packed SSEs, transform one of them using the packing axis by 1 to 3Å translation and 10 to 45 degrees rotation |
| 48 | helixpair_rotate_z_large_hinge | α-helix pair | 182671 | 1773 | 38758 | 117634 | 24506 | 1.0 | 21.2 | 64.4 | 13.4 | -69.5 | Locate two packed helices, rotate both 15 to 45 degrees around z axis of one of them |
| 49 | helixpair_rotate_z_large_no_hinge | α-helix pair | 182877 | 2062 | 39045 | 117310 | 24460 | 1.1 | 21.4 | 64.2 | 13.4 | -86.1 | Locate two packed helices, rotate one 15 to 45 degrees around z axis of the other one |
| 50 | helixdomain_flip_ext | α-helix domain | 60712 | 82 | 3509 | 12691 | 44430 | 0.1 | 5.8 | 20.9 | 73.2 | -93.8 | Locate a domain of helices, rotate them 180 degrees externally along a common x,y or z axis |
| 51 | helixdomain_flip_int | α-helix domain | 61382 | 134 | 4380 | 11968 | 44900 | 0.2 | 7.1 | 19.5 | 73.2 | -60.4 | Locate a domain of helices, rotate them 180 degrees internally along x,y or z axis |
| 52 | helixdomain_shuffle | α-helix domain | 243485 | 1030 | 53235 | 186850 | 2370 | 0.4 | 21.9 | 76.7 | 1.0 | -153.5 | Locate a domain of helices, swap locations of 1 or 2 pairs of helices |
| 53 | helixdomain_translate_large | α-helix domain | 61004 | 131 | 11713 | 48594 | 566 | 0.2 | 19.2 | 79.7 | 0.9 | -79.9 | Translate a domain of helices 2 to 6Å along any direction |
| 54 | helixdomain_rotate_large | α-helix domain | 61268 | 98 | 9559 | 51013 | 598 | 0.2 | 15.6 | 83.3 | 1.0 | -77.1 | Rotate a domain of helices 15 to 45 degrees along any axis |
| 55 | helixdomain_transform_large | α-helix domain | 60749 | 58 | 9034 | 51030 | 627 | 0.1 | 14.9 | 84.0 | 1.0 | -129.8 | Transform a domain of helices by 2 to 6Å translation and 15 to 45 degrees rotation along any axis |
| 56 | sheet_shuffle | β-sheet | 73863 | 648 | 14463 | 50071 | 8681 | 0.9 | 19.6 | 67.8 | 11.8 | -94.9 | Locate a sheet, swap locations of 1 or 2 pairs of strands |
| 57 | sheet_switch_strand | β-sheet | 18452 | 236 | 2291 | 6825 | 9100 | 1.3 | 12.4 | 37.0 | 49.3 | -163.4 | Remove a edge strand from a sheet and add it to another sheet |
| 58 | sheet_cycle | β-sheet | 18401 | 65 | 2625 | 11394 | 4317 | 0.4 | 14.3 | 61.9 | 23.5 | -144.8 | Locate a sheet, cycle the locations of 2 to 4 strands in the sheet by 1 to 3 positions |
| 59 | sheet_cycle_intact | β-sheet | 18409 | 90 | 2761 | 11260 | 4298 | 0.5 | 15.0 | 61.2 | 23.4 | -92.0 | Locate a sheet, cycle the locations of all strands in the sheet by 1 to 3 positions , while keeping relative parallel/antiparallel orientations intact |
| 60 | sheet_cycle_subset | β-sheet | 18390 | 102 | 5094 | 8901 | 4293 | 0.6 | 27.7 | 48.4 | 23.3 | -87.3 | Same as sheet_cycle, but instead of all strands, only moves 2 to 4 strands |
| 61 | sheet_cycle_subset_intact | β-sheet | 18402 | 141 | 5120 | 8805 | 4336 | 0.8 | 27.8 | 47.9 | 23.6 | -111.3 | Same as sheet_cyle_subset, but keeps the relative parallel/antiparallel orientations intact |
| 62 | sheet_divide | β-sheet | 18316 | 98 | 1513 | 7999 | 8706 | 0.5 | 8.3 | 43.7 | 47.5 | -84.1 | Locate a sheet of at least 4 strands and divide it to two sheets of at least 2 strands each and then translate one sheet away from up to 4Å in each direction |
| 63 | sheet_divide_sandwich | β-sheet | 18229 | 15 | 884 | 8751 | 8579 | 0.1 | 4.9 | 48.0 | 47.1 | -135.9 | Locate a sheet of at least 4 strands and divide it to two sheets of at least 2 strands each and then pack one of the new sheets against the other one in beta-sandwich form |
| 64 | sheet_flip_ext | β-sheet | 18214 | 127 | 6206 | 9704 | 2177 | 0.7 | 34.1 | 53.3 | 12.0 | -61.7 | Rotate all strands in a sheet externally along a common x, y or z axis |
| 65 | sheet_flip_int | β-sheet | 18052 | 238 | 4286 | 11351 | 2177 | 1.3 | 23.7 | 62.9 | 12.1 | -36.5 | Rotate all strands in a sheet internally along x, y or z axis |
| 66 | sheet_flip_int_sub | β-sheet | 18390 | 334 | 4705 | 11104 | 2247 | 1.8 | 25.6 | 60.4 | 12.2 | -41.6 | Rotate a subset of strands in a sheet internally along x,y or z axis |
| 67 | sheet_flip_int_sub_diff | β-sheet | 18484 | 282 | 4074 | 11891 | 2237 | 1.5 | 22.0 | 64.3 | 12.1 | -58.2 | Rotate a subset of strands in a sheet along different axes |
| 68 | sheet_pair_strands | β-sheet | 18338 | 158 | 1109 | 1842 | 15229 | 0.9 | 6.1 | 10.0 | 83.1 | -242.6 | Locate unpaired strands and pair them with each other, if there is only one unpaired strand, then add it to a sheet |
| 69 | sheet_register_shift | β-sheet | 18550 | 258 | 4067 | 9896 | 4329 | 1.4 | 21.9 | 53.4 | 23.3 | -43.4 | Shift the hydrogen bonding register of two strands in a sheet by a translation in the amoun of two residue lengths |
| 70 | sheet_register_shift_flip | β-sheet | 18235 | 536 | 4975 | 8513 | 4211 | 2.9 | 27.3 | 46.7 | 23.1 | -35.4 | Shift the hydrogen bonding register of two strands in a sheet by a translation in the amount of one residue length coupled with a 180 degrees rotation around x or y axis |
| 71 | sheet_translate_large | β-sheet | 18237 | 210 | 6887 | 10987 | 153 | 1.2 | 37.8 | 60.3 | 0.8 | -50.3 | Translate a sheet by 2 to 4Å along any axis |
| 72 | sheet_rotate_large | β-sheet | 18310 | 206 | 6233 | 11717 | 154 | 1.1 | 34.0 | 64.0 | 0.8 | -47.7 | Rotate a sheet by 15 to 45 degrees around any axis |
| 73 | sheet_transform_large | β-sheet | 18435 | 91 | 6147 | 12034 | 163 | 0.5 | 33.3 | 65.3 | 0.9 | -46.4 | Transform a sheet by 2 to 4Å translation and 15 to 45 degreess rotation |
| 74 | sheet_twist_large | β-sheet | 18379 | 1591 | 4648 | 7774 | 4366 | 8.7 | 25.3 | 42.3 | 23.8 | -59.4 | Adjust the twist angle of all strands in a sheet by up to 10 degrees rotations |

**Table S1: Moves used in BCL::Fold assembly protocol**
