# Supplementary material for: BCL::Fold - De Novo Prediction of Complex and Large Protein Topologies by Assembly of Secondary Structure Elements
Source: PLoS One. 2012 Nov 16;7(11):e49240. doi: 10.1371/journal.pone.0049240 (PMC3500284; doi:10.1371/journal.pone.0049240)
Supplement: Table S2 — Moves used in BCL::Fold refinement protocol. (DOCX) [file pone.0049240.s005.docx]

Table S2 lists all moves used in BCL::Fold refinement protocol along with the subcategory they belong to. This is followed by counts and percentages on minimization steps where each move was used along with what kind of Metropolis result these steps have led to; total number of steps used in(N_T_), number and percentage of improved steps(N_I_ and P_I_), accepted steps (N_A_ and P_A_), rejected steps (N_R_ and P_R_), skipped steps(N_S_ and P_S_). This is followed by Δ_MEAN,_ which represents the average energy decrease in the energy from the last improved model for cases where the move has led to an improved step. The last column gives a short description of what each move does.

| **Move#** | **Move** | **Type** | **N_total_** | **N_improved_** | **N_accepted_** | **N_rejected_** | **N_skipped_** | **%_improved_** | **%_accepted_** | **%_rejected_** | **%_skipped_** | **Δ_mean_** | **description** |
| --- | --- | --- | --- | --- | --- | --- | --- | --- | --- | --- | --- | --- | --- |
| 1 | sse_bend_ramachandran | SSE | 6957 | 588 | 1426 | 4943 | 0 | 8.5 | 20.5 | 71.1 | 0.0 | -10.2 | Change phi/psi angles for a random residue using Ramachandran statistics |
| 2 | sse_bend_random_large | SSE | 7010 | 632 | 1579 | 4799 | 0 | 9.0 | 22.5 | 68.5 | 0.0 | -14.3 | Change phi/psi angles for a random residue by 0 to 20 degrees |
| 3 | sse_bend_random_small | SSE | 6846 | 1610 | 2344 | 2892 | 0 | 23.5 | 34.2 | 42.2 | 0.0 | -9.6 | Change phi/psi angles for a random residue by 0 to 5 degrees |
| 4 | sse_resize_cterm | SSE | 10362 | 622 | 1524 | 6854 | 1362 | 6.0 | 14.7 | 66.2 | 13.1 | -51.0 | Extend/shrink a random SSE by 1 to 3 residues from C terminal end |
| 5 | sse_resize_nterm | SSE | 10416 | 647 | 1495 | 6913 | 1361 | 6.2 | 14.4 | 66.4 | 13.1 | -47.8 | Extend/shrink a random SSE by 1 to 3 residues from N terminal end |
| 6 | sse_rotate_small | SSE | 20599 | 829 | 3641 | 16129 | 0 | 4.0 | 17.7 | 78.3 | 0.0 | -19.1 | Rotate an SSE by up to 15 degrees in any direction |
| 7 | sse_rotate_x_small | SSE | 7072 | 608 | 1603 | 4861 | 0 | 8.6 | 22.7 | 68.7 | 0.0 | -10.6 | Rotate an SSE by up to 15 degrees around X axis |
| 8 | sse_rotate_y_small | SSE | 6784 | 782 | 1693 | 4309 | 0 | 11.5 | 25.0 | 63.5 | 0.0 | -13.3 | Rotate an SSE by up to 15 degrees around Y axis |
| 9 | sse_rotate_z_small | SSE | 6869 | 1357 | 2737 | 2775 | 0 | 19.8 | 39.9 | 40.4 | 0.0 | -5.7 | Rotate an SSE by up to 15 degrees around Z axis |
| 10 | sse_translate_small | SSE | 20812 | 650 | 3647 | 16515 | 0 | 3.1 | 17.5 | 79.4 | 0.0 | -21.3 | Translate an SSE up to 2Å along any direction |
| 11 | sse_translate_x_small | SSE | 6889 | 891 | 1947 | 4051 | 0 | 12.9 | 28.3 | 58.8 | 0.0 | -9.3 | Translate an SSE up to 2Å along X axis |
| 12 | sse_translate_y_small | SSE | 6813 | 654 | 1749 | 4410 | 0 | 9.6 | 25.7 | 64.7 | 0.0 | -14.6 | Translate an SSE up to 2Å along Y axis |
| 13 | sse_translate_z_small | SSE | 6823 | 1139 | 2552 | 3132 | 0 | 16.7 | 37.4 | 45.9 | 0.0 | -4.2 | Translate an SSE up to 2Å along Z axis |
| 14 | sse_transform_small | SSE | 41527 | 552 | 5518 | 35457 | 0 | 1.3 | 13.3 | 85.4 | 0.0 | -24.5 | Transform an SSE in any direction by up to 2Å translation and 15 degree rotation |
| 15 | helix_translate_xy_small | α-helix | 31385 | 1662 | 7802 | 21768 | 153 | 5.3 | 24.9 | 69.4 | 0.5 | -10.2 | Translate an helix up to 2Å along x axis and up to 2Å along y axis |
| 16 | helix_translate_z_small | α-helix | 31225 | 5828 | 13065 | 12155 | 177 | 18.7 | 41.8 | 38.9 | 0.6 | -4.2 | Translate an helix up to 2Å along z axis |
| 17 | helix_rotate_xy_small | α-helix | 31087 | 1782 | 7151 | 21993 | 161 | 5.7 | 23.0 | 70.8 | 0.5 | -12.7 | Rotate an helix up to 15 degrees around x axis and up to 15 degrees around y axis |
| 18 | helix_rotate_z_small | α-helix | 31395 | 6854 | 14695 | 9684 | 162 | 21.8 | 46.8 | 30.9 | 0.5 | -3.5 | Rotate an helix up to 15 degrees around z axis |
| 19 | helix_transform_xy_small | α-helix | 31406 | 850 | 6001 | 24355 | 200 | 2.7 | 19.1 | 77.6 | 0.6 | -16.9 | Transform a helix by up to 2A translation and up to 15 degrees rotation in z axis |
| 20 | helix_transform_z_small | α-helix | 31472 | 3535 | 10795 | 16986 | 156 | 11.2 | 34.3 | 54.0 | 0.5 | -6.6 | Transform a helix by up to 2A translation and up to 15 degrees rotation in z axis |
| 21 | strand_translate_z_small | β-strand | 143507 | 18861 | 47832 | 74976 | 1838 | 13.1 | 33.3 | 52.3 | 1.3 | -3.5 | Translate a strand up to 2Å along z axis |
| 22 | ssepair_translate_small | SSE pair | 36292 | 1763 | 4964 | 9877 | 19688 | 4.9 | 13.7 | 27.2 | 54.3 | -8.7 | Locate two packed SSEs, translate one of them up to 3Å along the packing axis |
| 23 | ssepair_translate_no_hinge_small | SSE pair | 36071 | 423 | 2970 | 13140 | 19538 | 1.2 | 8.2 | 36.4 | 54.2 | -19.0 | Locate two packed SSEs, translate one them up to 2Å in any axis of the other one |
| 24 | ssepair_rotate_small | SSE pair | 35925 | 1234 | 4001 | 11176 | 19514 | 3.4 | 11.1 | 31.1 | 54.3 | -8.7 | Locate two packed SSEs, rotate one of them up to 15 degrees around the packing axis |
| 25 | ssepair_transform_small | SSE pair | 36493 | 817 | 3549 | 12164 | 19963 | 2.2 | 9.7 | 33.3 | 54.7 | -13.1 | Locate two packed SSEs, transform one of them using the packing axis up to 1Å translation and up to 15 degrees rotation |
| 26 | helixpair_rotate_z_small_hinge | α-helix pair | 94151 | 4341 | 21163 | 59044 | 9603 | 4.6 | 22.5 | 62.7 | 10.2 | -6.1 | Locate two packed helices, rotate both up to 15 degrees around z axis of one of them |
| 27 | helixpair_rotate_z_small_no_hinge | α-helix pair | 93704 | 4443 | 21179 | 58624 | 9458 | 4.7 | 22.6 | 62.6 | 10.1 | -6.6 | Locate two packed helices, rotate one up to 15 degrees around z axis of the other one |
| 28 | helixdomain_translate_small | α-helix domain | 93796 | 975 | 15568 | 77253 | 0 | 1.0 | 16.6 | 82.4 | 0.0 | -17.3 | Translate a domain of helices up to 2Å along any direction |
| 29 | helixdomain_rotate_small | β-sheet | 93584 | 430 | 12326 | 80828 | 0 | 0.5 | 13.2 | 86.4 | 0.0 | -19.9 | Rotate a domain of helices up to 15 degrees along any axis |
| 30 | helixdomain_transform_small | β-sheet | 93535 | 32 | 9136 | 84367 | 0 | 0.0 | 9.8 | 90.2 | 0.0 | -23.4 | Transform a domain of helices by up to 2Å translation and up to 30 degrees rotation |
| 31 | sheet_register_fix | β-sheet | 41478 | 451 | 5838 | 26748 | 8441 | 1.1 | 14.1 | 64.5 | 20.4 | -9.8 | Fix the hydrogen bonding pattern of a located sheet by applying small translations |
| 32 | sheet_translate_small | β-sheet | 41440 | 1088 | 13723 | 26523 | 106 | 2.6 | 33.1 | 64.0 | 0.3 | -15.9 | Translate a sheet by up to 2 Å along any axis |
| 33 | sheet_rotate_small | β-sheet | 41686 | 734 | 12710 | 28154 | 88 | 1.8 | 30.5 | 67.5 | 0.2 | -26.6 | Rotate a sheet by up to 15 degrees around any axis |
| 34 | sheet_transform_small | β-sheet | 41375 | 311 | 11915 | 29045 | 104 | 0.8 | 28.8 | 70.2 | 0.3 | -29.9 | Transform a sheet by up to 2 Å translation and up to 15 degrees rotation |
| 35 | sheet_twist_small | β-sheet | 41713 | 5153 | 8710 | 19478 | 8372 | 12.4 | 20.9 | 46.7 | 20.1 | -11.6 | Adjust the twist angle of all strands in a sheet by up to 2 degrees rotations |

**Table S2: Moves used in BCL::Fold refinement protocol**
