# Supplementary material for: BCL::Fold - De Novo Prediction of Complex and Large Protein Topologies by Assembly of Secondary Structure Elements
Source: PLoS One. 2012 Nov 16;7(11):e49240. doi: 10.1371/journal.pone.0049240 (PMC3500284; doi:10.1371/journal.pone.0049240)
Supplement: Table S3 — Weightset for the energy function in BCL::Fold. (DOCX) [file pone.0049240.s006.docx]

Table S3 summarizes scores and weights in the energy function in BCL::Fold; amino acid clash score (aa_clash), amino acid distance score (aa_dist), amino acid environment potential and the counterpart for the unfolded fraction of the model (aa_neigh & aa_neigh_unf), SSE clash score (sse_clash), SSE packing score (sse_pack), β-strand pairing score (strand_pair), loop score (loop), loop closure score (loop_closure), radius of gyration score (rgyr), contact order score (co) contact order score, SSE prediction scores and the counterparts for the unfolded fraction of the model using methods JUFO (sse_JUFO & ss_JUFO_unf and PSIPRED (ss_PSIPRED & ss_PSIPRED_unf).

| **energy function** | **weight** |
| --- | --- |
| aa_clash | 500.00 |
| aa_dist | 0.35 |
| aa_neigh | 50.00 |
| aa_neigh_unf | 50.00 |
| sse_clash | 500.00 |
| sse_pack | 8.00 |
| strand_pair | 20.00 |
| loop | 10.00 |
| loop_closure | 500.00 |
| rgyr | 5.00 |
| co | 0.50 |
| ss_JUFO* | 5.00 |
| ss_JUFO_unf* | 5.00 |
| ss_PSIPRED* | 20.00 |
| ss_SIPRED_unf* | 20.00 |

**Table S3: Weightset for the energy function in BCL::Fold**
