# Supplementary material for: BCL::Fold - De Novo Prediction of Complex and Large Protein Topologies by Assembly of Secondary Structure Elements
Source: PLoS One. 2012 Nov 16;7(11):e49240. doi: 10.1371/journal.pone.0049240 (PMC3500284; doi:10.1371/journal.pone.0049240)
Supplement: Table S4 — Residue coverage of BCL::Fold models relative to the number of amino acids in the native protein structure. (DOCX) [file pone.0049240.s007.docx]

Table S4 reports the coverage of modeled residues in BCL::Fold SSE-only models. The number of amino acids in the native structure (N_aa_) and the number of amino acids in SSEs (after filtering by size) (N_SSE_) are used to calculate the relative coverage in %. The coverage relative to the total number of amino acids (COV) is calculated for BCL::Fold models using native SSEs (COV_N-SSE_) and predicted SSEs (COV_P-SSE_). The coverage relative to the number of amino acids in SSEs of the native structure (SSE-COV) is also shown for BCL::Fold models using native SSEs (SSE-COV_N-SSE_) and SSEs from a predicted pool (SSE-COV_P-SSE_). The latter is typically above 100%, which is attributed to the over-prediction of SSE length which generates longer SSE definitions, that just cross the SSE-length criteria that is used for filtering short SSEs.

| **PDB id** | **N_aa_** | **N_sse_** | **COV_N-sse_** | **COV_P-sse_** | **SSE-COV_N-sse_** | **SSE-COV_P-sse_** |
| --- | --- | --- | --- | --- | --- | --- |
| 1BGCA | 174 | 108 | 62.1 ± 0.0 | 64.4 ± 2.9 | 100.0 ± 0.0 | 103.7 ± 4.6 |
| 1EYHA | 144 | 107 | 73.2 ± 2.4 | 74.4 ± 2.4 | 98.5 ± 3.2 | 100.2 ± 3.2 |
| 1FQIA | 147 | 90 | 60.4 ± 1.7 | 65.4 ± 2.8 | 98.6 ± 2.8 | 106.8 ± 4.6 |
| 1GAKA | 141 | 96 | 68.1 ± 0.0 | 67.1 ± 2.9 | 100.0 ± 0.0 | 98.5 ± 4.2 |
| 1GYUA | 140 | 63 | 45.0 ± 0.0 | 50.8 ± 3.0 | 100.0 ± 0.0 | 112.9 ± 6.7 |
| 1IAPA | 211 | 123 | 58.2 ± 0.6 | 62.2 ± 1.9 | 99.9 ± 1.0 | 106.7 ± 3.2 |
| 1ICXA | 155 | 103 | 66.1 ± 1.4 | 70.5 ± 2.7 | 99.4 ± 2.1 | 106.2 ± 4.1 |
| 1J27A | 102 | 76 | 74.5 ± 0.2 | 75.4 ± 3.3 | 100.0 ± 0.2 | 101.2 ± 4.5 |
| 1JL1A | 155 | 97 | 60.5 ± 3.5 | 58.4 ± 2.5 | 96.6 ± 5.6 | 93.3 ± 4.0 |
| 1LKIA | 180 | 113 | 62.8 ± 0.1 | 54.7 ± 2.9 | 100.0 ± 0.1 | 87.1 ± 4.6 |
| 1LMIA | 131 | 63 | 47.9 ± 1.0 | 42.5 ± 3.9 | 99.5 ± 2.1 | 88.4 ± 8.1 |
| 1OXJA | 173 | 108 | 62.4 ± 0.3 | 67.6 ± 3.0 | 100.0 ± 0.4 | 108.3 ± 4.8 |
| 1OZ9A | 150 | 101 | 65.8 ± 1.1 | 64.0 ± 3.2 | 97.8 ± 1.6 | 95.1 ± 4.7 |
| 1PBVA | 195 | 128 | 65.6 ± 0.0 | 65.8 ± 1.5 | 100.0 ± 0.0 | 100.3 ± 2.3 |
| 1PKOA | 139 | 58 | 41.7 ± 0.0 | 54.4 ± 3.6 | 100.0 ± 0.0 | 130.3 ± 8.6 |
| 1Q5ZA | 177 | 77 | 43.5 ± 0.0 | 52.4 ± 2.9 | 100.0 ± 0.0 | 120.5 ± 6.6 |
| 1RJ1A | 151 | 113 | 74.8 ± 0.2 | 77.7 ± 2.5 | 100.0 ± 0.2 | 103.8 ± 3.3 |
| 1T3YA | 141 | 83 | 58.7 ± 1.0 | 60.9 ± 2.8 | 99.7 ± 1.6 | 103.4 ± 4.8 |
| 1TP6A | 128 | 94 | 72.1 ± 1.7 | 69.7 ± 3.6 | 98.2 ± 2.3 | 94.9 ± 4.9 |
| 1TQGA | 105 | 88 | 83.8 ± 0.0 | 79.8 ± 3.5 | 100.0 ± 0.0 | 95.2 ± 4.1 |
| 1TZVA | 142 | 97 | 68.3 ± 0.0 | 70.7 ± 2.3 | 100.0 ± 0.0 | 103.5 ± 3.4 |
| 1UAIA | 224 | 114 | 50.8 ± 0.7 | 50.2 ± 2.4 | 99.8 ± 1.3 | 98.7 ± 4.7 |
| 1ULRA | 88 | 55 | 65.9 ± 0.1 | 71.6 ± 3.1 | 105.5 ± 0.1 | 114.6 ± 4.9 |
| 1VINA | 268 | 156 | 58.2 ± 0.2 | 66.8 ± 2.0 | 100.0 ± 0.4 | 114.8 ± 3.4 |
| 1X91A | 153 | 113 | 73.9 ± 0.0 | 73.9 ± 2.7 | 100.0 ± 0.0 | 100.1 ± 3.7 |
| 1XAKA | 83 | 38 | 45.8 ± 0.0 | 47.6 ± 4.8 | 100.0 ± 0.1 | 104.0 ± 10.4 |
| 1XKRA | 206 | 147 | 69.0 ± 1.2 | 68.4 ± 2.4 | 96.7 ± 1.7 | 95.9 ± 3.4 |
| 1XQOA | 256 | 162 | 62.6 ± 1.5 | 63.7 ± 2.6 | 98.9 ± 2.4 | 100.6 ± 4.1 |
| 1Z3XA | 238 | 129 | 53.3 ± 1.2 | 58.0 ± 1.8 | 98.3 ± 2.2 | 107.1 ± 3.4 |
| 2AP3A | 199 | 156 | 78.4 ± 0.0 | 71.4 ± 4.7 | 100.0 ± 0.0 | 91.1 ± 6.0 |
| 2BK8A | 97 | 47 | 48.5 ± 0.0 | 63.6 ± 4.3 | 100.0 ± 0.0 | 131.3 ± 8.9 |
| 2CWRA | 103 | 60 | 57.9 ± 1.3 | 54.7 ± 3.7 | 99.5 ± 2.3 | 93.8 ± 6.3 |
| 2EJXA | 139 | 107 | 75.1 ± 3.3 | 65.8 ± 3.7 | 97.5 ± 4.3 | 85.5 ± 4.7 |
| 2F1SA | 186 | 115 | 60.9 ± 1.5 | 65.3 ± 2.4 | 98.4 ± 2.5 | 105.6 ± 3.9 |
| 2FC3A | 124 | 80 | 63.2 ± 1.9 | 70.8 ± 3.6 | 98.0 ± 2.9 | 109.7 ± 5.5 |
| 2FM9A | 215 | 153 | 71.0 ± 1.0 | 74.1 ± 3.1 | 99.8 ± 1.4 | 104.1 ± 4.3 |
| 2FRGP | 106 | 64 | 60.2 ± 1.0 | 63.3 ± 4.7 | 99.6 ± 1.7 | 104.9 ± 7.8 |
| 2GKGA | 127 | 80 | 59.8 ± 4.0 | 63.8 ± 2.6 | 94.9 ± 6.4 | 101.3 ± 4.2 |
| 2HUJA | 140 | 99 | 70.7 ± 0.0 | 66.6 ± 2.7 | 100.0 ± 0.0 | 94.2 ± 3.8 |
| 2IU1A | 208 | 126 | 60.5 ± 0.6 | 64.1 ± 1.8 | 99.9 ± 0.9 | 105.8 ± 2.9 |
| 2JLIA | 123 | 69 | 56.0 ± 0.6 | 71.4 ± 3.2 | 99.9 ± 1.0 | 127.3 ± 5.8 |
| 2LISA | 136 | 91 | 66.9 ± 0.0 | 63.4 ± 2.6 | 100.0 ± 0.0 | 94.7 ± 3.9 |
| 2OF3A | 266 | 202 | 72.4 ± 2.6 | 73.2 ± 3.0 | 95.4 ± 3.4 | 96.4 ± 4.0 |
| 2OSAA | 202 | 124 | 61.4 ± 0.2 | 60.5 ± 2.4 | 100.0 ± 0.3 | 98.6 ± 3.9 |
| 2QZQA | 152 | 63 | 41.4 ± 0.1 | 50.4 ± 3.1 | 100.0 ± 0.3 | 121.5 ± 7.5 |
| 2R0SA | 285 | 165 | 55.0 ± 3.5 | 54.0 ± 1.9 | 95.0 ± 6.1 | 93.3 ± 3.3 |
| 2RB8A | 104 | 46 | 44.2 ± 0.0 | 51.5 ± 3.5 | 100.0 ± 0.0 | 116.5 ± 7.8 |
| 2RCIA | 204 | 126 | 60.5 ± 1.5 | 63.4 ± 3.3 | 97.9 ± 2.5 | 102.7 ± 5.3 |
| 2V75A | 104 | 65 | 62.5 ± 0.0 | 74.0 ± 3.6 | 100.0 ± 0.0 | 118.5 ± 5.8 |
| 2VQ4A | 106 | 54 | 49.8 ± 2.0 | 62.2 ± 3.2 | 97.7 ± 4.0 | 122.1 ± 6.2 |
| 2WJ5A | 101 | 42 | 41.6 ± 0.0 | 55.4 ± 3.6 | 100.0 ± 0.0 | 133.2 ± 8.6 |
| 2WWEA | 127 | 69 | 52.9 ± 1.9 | 58.8 ± 2.9 | 97.4 ± 3.5 | 108.3 ± 5.2 |
| 2YV8A | 164 | 79 | 48.2 ± 0.1 | 51.4 ± 2.7 | 100.0 ± 0.3 | 106.7 ± 5.5 |
| 2YXFA | 100 | 46 | 46.0 ± 0.0 | 54.9 ± 4.3 | 100.0 ± 0.0 | 119.3 ± 9.2 |
| 2YYOA | 171 | 66 | 38.5 ± 0.4 | 49.6 ± 2.7 | 99.8 ± 1.1 | 128.6 ± 7.1 |
| 2ZCOA | 293 | 205 | 68.1 ± 1.9 | 72.4 ± 2.3 | 97.4 ± 2.7 | 103.5 ± 3.3 |
| 3B5OA | 244 | 169 | 69.3 ± 0.0 | 73.8 ± 3.3 | 100.0 ± 0.0 | 106.6 ± 4.8 |
| 3CTGA | 129 | 68 | 51.2 ± 2.8 | 60.0 ± 3.1 | 97.0 ± 5.4 | 113.9 ± 6.0 |
| 3CX2A | 108 | 53 | 49.1 ± 0.0 | 64.0 ± 4.1 | 100.0 ± 0.0 | 130.4 ± 8.3 |
| 3FH2A | 146 | 100 | 68.5 ± 0.1 | 69.8 ± 1.8 | 100.0 ± 0.2 | 101.9 ± 2.6 |
| 3FHFA | 214 | 147 | 67.2 ± 3.0 | 67.6 ± 2.8 | 97.8 ± 4.4 | 98.5 ± 4.1 |
| 3FRRA | 191 | 141 | 73.6 ± 0.8 | 70.4 ± 1.6 | 99.7 ± 1.1 | 95.4 ± 2.1 |
| 3HVWA | 176 | 109 | 57.7 ± 3.3 | 68.6 ± 2.7 | 93.2 ± 5.4 | 110.8 ± 4.3 |
| 3IV4A | 112 | 77 | 66.6 ± 1.9 | 65.6 ± 2.8 | 96.9 ± 2.7 | 95.4 ± 4.1 |
| 3NE3B | 130 | 81 | 60.7 ± 0.7 | 71.0 ± 4.1 | 97.4 ± 1.1 | 113.9 ± 6.5 |
| 3OIZA | 99 | 63 | 63.5 ± 0.9 | 71.1 ± 3.6 | 99.8 ± 1.4 | 111.7 ± 5.7 |
| avg | 160 | 99 | 60.5 | 64.0 | 99.1 | 106.0 |
| std | 51 | 38 | 10.3 | 8.2 | 1.7 | 11.3 |

**Table S4: Residue coverage of BCL::Fold models relative to the number of amino acids in the native protein structure**
