# Supplementary material for: BCL::Fold - De Novo Prediction of Complex and Large Protein Topologies by Assembly of Secondary Structure Elements
Source: PLoS One. 2012 Nov 16;7(11):e49240. doi: 10.1371/journal.pone.0049240 (PMC3500284; doi:10.1371/journal.pone.0049240)
Supplement: Table S5 — Movie file names for different proteins and minimization stages with information about the model. (DOCX) [file pone.0049240.s008.docx]

Table S5 lists different movies that demonstrate the BCL::Fold assembly procedure available as supporting information. The minimizations with native SSE definitions of models with the best RMSD100 after stage 1 were selected. One trajectory was selected for each protein class (α-helical, β-sheet and αβ) and each stage has a separate movie.

| Pdb | | 1TQGA | 2RB8A | 3IV4A |
| --- | --- | --- | --- | --- |
| Class | | α | β | αβ |
| #α-helix | | 5 | 0 | 4 |
| #β-strand | | 0 | 6 | 5 |
| RMSD100 | BCL_N-SSE_ | 2.40 Å | 5.80 Å | 3.34 Å |
|  | BCL_N_ | 3.30 Å | 8.48 Å | 4.68 Å |
|  | Rosetta | 2.49 Å | 7.86 Å | 3.98 Å |
| Stage | Assembly | Movie_S1.mp4 | Movie_S6.mp4 | Movie_S11.mp4 |
|  | Refinement | Movie_S2.mp4 | Movie_S7.mp4 | Movie_S12.mp4 |
|  | Loop grow | Movie_S3.mp4 | Movie_S8.mp4 | Movie_S13.mp4 |
|  | Loop close | Movie_S4.mp4 | Movie_S9.mp4 | Movie_S14.mp4 |
|  | Loop force close | Movie_S5.mp4 | Movie_S10.mp4 | Movie_S15.mp4 |

**Table S5 Movie file names for different proteins and minimization stages**

The movie shows the pdb structure in transparent grey. The models are colored by amino acid sequence position and are superimposed onto the pdb structure. On the right, a table with the current weighted scores and their sum is displayed. Rmsd100 and cr12 quality values of the current model are shown as well. On the lower right, the current Metropolis condition is displayed as a traffic light.
