# Supplementary material for: BCL::Fold - De Novo Prediction of Complex and Large Protein Topologies by Assembly of Secondary Structure Elements
Source: PLoS One. 2012 Nov 16;7(11):e49240. doi: 10.1371/journal.pone.0049240 (PMC3500284; doi:10.1371/journal.pone.0049240)
Supplement: Appendix S1 — BCL::Fold command line usage and file formats. (DOCX) [file pone.0049240.s009.docx]

**Appendix S1 BCL::Fold command line usage and sample trajectories as movies**

BCL::Fold requires the availability of a fasta file for the protein of interest accompanied by the corresponding secondary structure predictions from PSIPRED and JUFO as well as a SSE pool file which can be generated using BCL::SSEPool application.

SSEPool file for 1J27 is shown below. Since it includes predictions from both PSIPRED and JUFO, it contains overlapping definitions.

bcl::assemble::SSEPool

SHEET 1 LYS A 2 GLU A 13

HELIX 2 2 LEU A 19 ARG A 36 1 18

HELIX 3 3 LYS A 22 ALA A 35 1 14

SHEET 4 PHE A 37 SER A 40

SHEET 5 VAL A 39 LEU A 44

SHEET 6 ALA A 42 LEU A 44

SHEET 7 TYR A 52 GLY A 61

HELIX 8 8 PRO A 64 GLU A 80 1 17

HELIX 9 9 GLU A 68 GLU A 80 1 13

HELIX 10 10 PHE A 84 GLU A 97 1 14

SHEET 11 GLN A 85 LEU A 98

END

Below is a sample command line used for running BCL::Fold which also corresponds to the command lines used for the BCL::Fold minimizations with predicted SSE pools presented in this manuscript.

./bcl.exe Fold -native input/1J27A.pdb -pool_separate 1 -pool_min_sse_lengths 5 3 -quality RMSD GDT_TS -superimpose RMSD -message_level Critical -function_cache -sspred JUFO PSIPRED -sspred_path_prefix input/ 1J27A -stages_read input/stages.txt -pool input/1J27A.pool -loop_closure_threshold 0.1 -loop_rama_mutate_prob 0.0 -ccd_fraction [0.5,1.0] -nmodels 5 -prefix out/ -random_seed 1

The commandline above reads in necessary input files from a directory named “input” and outputs the pdbs of the predicted models to an output directory named “out”. The “input” directory contains the following files

- *1J27A.pdb* – PDB file needed for RMSD and GDT_TS calculations. If a pdb is not available as in a blind experiment case, then instead of “-native input/1J27A.pdb” flag, “-fasta 1J27A.fasta” can be used.
- *1J27A.pool* – Pool file which includes predicted SS predictions
- *1J27A.psipred_ss2* – psipred predictions for 1J27
- *1J27A.jufo_ss* – Jufo predictions for 1J27
- *stages*.txt – Stage file which include the necessary options for defining the various stages in the minimizations.

The stages file defines the stages to be used in the minimizations. A sample stage file used for the data presented is shown below:

NUMBER_CYCLES 1

STAGE Stage_assembly

FOLD_PROTOCOLS Default Assembly

SCORE_PROTOCOLS Default

SCORE_WEIGHTSET_FILE assembly.scoreweights

MUTATE_PROTOCOLS Default Assembly

NUMBER_ITERATIONS 5000 1000

STAGE_END

STAGE Stage_refinement

FOLD_PROTOCOLS Default Refinement

SCORE_PROTOCOLS Default

SCORE_WEIGHTSET_FILE assembly.scoreweights

MUTATE_PROTOCOLS Default Refinement

NUMBER_ITERATIONS 2000 400

PRINT_END_MODEL true

STAGE_END

STAGE Stage_loop_grow

FOLD_PROTOCOLS Default LoopCoordinateAdd

SCORE_WEIGHTSET_FILE loop_add_coordinates.scoreweights

NUMBER_ITERATIONS 3500 600

MODIFY_START_MODEL true

STAGE_END

STAGE Stage_close

FOLD_PROTOCOLS Default LoopClose

SCORE_WEIGHTSET_FILE loop_close.scoreweights

NUMBER_ITERATIONS 5000 250

MODIFY_START_MODEL true

STAGE_END

STAGE Stage_force_close

FOLD_PROTOCOLS Default LoopClose

SCORE_WEIGHTSET_FILE loop_close_force.scoreweights

NUMBER_ITERATIONS 2500 125

MODIFY_START_MODEL true

STAGE_END

The first two main stages assembly and refinement use the same score weightset as defined in the Table S3. These are followed by CCD-based loop building protocol divided into 3 short stages.
